# Improving Azo Dye Decolorization Performance and Halotolerance of *Pichia occidentalis* A2 by Static Magnetic Field and Possible Mechanisms Through Comparative Transcriptome Analysis

**DOI:** 10.3389/fmicb.2020.00712

**Published:** 2020-05-05

**Authors:** Xiaohan Wang, Yumeng Wang, Shuxiang Ning, Shengnan Shi, Liang Tan

**Affiliations:** School of Life Sciences, Liaoning Normal University, Dalian, China

**Keywords:** azo dye, *Pichia occidentalis* A2, halotolerance, static magnetic field (SMF), comparative transcriptome analysis

## Abstract

A halotolerant yeast, *Pichia occidentalis* A2, was recently isolated that can decolorize various azo dyes. The azo dye decolorization performance of this strain was characterized, including the degradation pathway and detoxification effects of this yeast. Additionally, the effect of static magnetic field (SMF) on this decolorization process was investigated. Activities of key enzymes were analyzed to estimate the change of metabolic activity. Furthermore, possible mechanisms were analyzed through detecting differentially expressed genes between yeast A2 in the absence and presence of SMF. The results indicated that yeast A2 displayed the optimal decolorization performance when the concentrations (in g/L) of glucose, (NH_4_)_2_SO_4_, yeast extract, and NaCl were 4.0, 1.0, 0.1, and ≤30.0, respectively. Meanwhile, the optimal rotation speed, temperature, and pH were 160 rpm, 30°C, and 5.0, respectively. Acid Red B was decolorized and detoxified by yeast A2 through successive steps, including cleavage of the naphthalene–amidine bond, reductive deamination, oxidative deamination/desulfurization, open-loop of hydroxy-substituted naphthalene, and tricarboxylic acid cycle. The dye decolorization efficiency and halotolerance of yeast A2 were enhanced by 206.3 mT SMF. The activities of manganese peroxidase, and laccase were elevated 1.37- and 1.16-fold by 206.3 mT SMF, but lignin peroxidase activity showed little change. It was suggested from the transcriptome sequence that the enhanced halotolerance might be related to the upregulated genes encoding the enzymes or functional proteins related to intracellular synthesis and accumulation of glycerol.

## Introduction

Hypersaline wastewater typically contains a variety of substances, including salts (at least 1%, *m*/*v*), organic matter, heavy materials, and radioactive materials (Pendashteh et al., 2011). Wastewater from the printing and dyeing industry is a representative type of hypersaline wastewater and usually contains a large amount of unused dyes. Azo dyes are the most widely used type worldwide (Tony et al., 2009). The discharge of azo dyes into water will consume dissolved oxygen, destroy the ecological balance of water, and can adversely affect the survival of aquatic organisms.

Biological processes are generally acknowledged as highly efficient, low-cost, and environment-friendly methods to eliminate environmental pollutants (Pearce et al., 2003). Different bacteria and fungi are tried to decolorize azo compounds. Bacteria can degrade diverse organics and possess strong adaptability to complex environments. However, decolorization intermediates (especially aromatic amines) cannot be used or further degraded by most bacteria due to a lack of the required metabolic enzymes (Qu et al., 2010). Azo dyes can also be effectively decolorized by Basidiomycota and Ascomycota fungi through bioabsorption and/or biodegradation (Kalmis et al., 2008; Arora et al., 2011). Furthermore, many fungi can detoxify azo dyes by decomposing the toxic intermediates using their non-specific oxidoreductive enzymes (Tan et al., 2013). For instance, strains of *Thielavia* sp. and *Hanerochaete chrysosporium* can efficiently degrade phenols (Lu et al., 2009; Mtibaà et al., 2020). Therefore, it is expected that fungi could be effectively applied for the deep purification of recalcitrant organic pollutants.

Previous studies reported that the application of static magnetic field (SMF) can impact microbial metabolism, enzyme activity, and cell membrane permeability to improve biological treatment effectiveness (Kovacs et al., 1997; Liu et al., 2008). As reported, SMFs of strong intensity (1–5 T) and ultra-strong intensity (higher than 5 T) generally exert inhibitory effects on organisms, but SMFs of moderate intensity always exert positive effects (Filipič et al., 2012; Łebkowska et al., 2013). Moderate-intensity SMFs have been reported to improve the removal efficiency of oil, phenol, and formaldehyde by single strains or microbial communities to degrade organic pollutants (Łebkowska et al., 2013; Křiklavová et al., 2014; Ren et al., 2018). An effect of SMF to improve the electrogenesis capacity of microbial fuel cells has also been confirmed (Yin et al., 2013). Furthermore, enhancement of microbial tolerance to specific conditions such as low temperature and high osmotic pressure was also observed through exposure to SMFs (Niu et al., 2014; Shao et al., 2019). All these results suggest that the existing biological treatment processes can be further improved by applying external SMF. However, little is known about the possible mechanisms of SMF action at the molecular level.

The purpose of this study was to investigate the effect of moderate-intensity SMF on a newly isolated halotolerant yeast strain for the aerobic biodegradation of azo dyes under high-salinity conditions. The influences of SMF on dye decolorization, cell growth, and resistance to high osmotic environment of the strain were studied. Finally, possible mechanisms of the effect of SMF were analyzed by comparative transcriptome sequencing.

## Materials and Methods

### Reagents

Six azo dyes were used, including Acid Red B, Reactive Green KE-4BD, Reactive Yellow 3RS, Reactive Brilliant Red K-2G, Acid Brilliant Scarlet GR, and Reactive Brilliant Red X-3B, abbreviated as ARB, KE-4BD, 3RS, K-2G, GR, and X-3B, respectively. The chemical structures and the characteristic absorption wavelengths of the six dyes are shown in Supplementary Table S1. Other biochemical and chemical reagents were purchased from Sangon Biotech Co., Ltd. and J&K Chemical Ltd. (both are located in Shanghai, China), respectively.

### Isolation, Identification, and Characterization of the Halotolerant Yeast

The yeast pure culture was isolated from sea mud sampled from a coastal sewage outfall of Dalian, China (121.57° E and 38.88° N) after acclimatization for 30 days. The culture medium contains (in g/L): glucose, 4.0; (NH_4_)_2_SO_4_, 1.0; yeast extract, 0.1; K_2_HPO_4_, 1.0; MgSO_4_×7H_2_O, 0.5; selected azo dye, 0.02–0.05; and NaCl, 30.0 (adjusted to pH 5.0–6.0). The medium was sterilized at 115°C for 15 min before use. The pure culture was isolated and purified through the spread-plate method, then was identified through 26S ribosomal DNA (rDNA) sequence analysis with the polymerase chain reaction (PCR) primers of “NL1 (GCATATCAATAAGCG GAGGAAAAG) and NL2 (GGTCCGTGTTTCAAGACGG)” for amplifying the D1/D2 region of 26S rDNA genes of yeast (Tan et al., 2013, 2016). Then, growth and environmental factors, including the concentrations of glucose (0–10.0 g/L), (NH_4_)_2_SO_4_ (0–1.0 g/L), yeast extract (for supplying the vitamin mixture, 0–0.3 g/L), and NaCl (0–150.0 g/L), the rotation speed (0–200 rpm), temperature (20–40°C), and pH (3.0–10.0), were optimized through batch tests in 250-mL flasks. The dye which was decolorized with the highest speed was used as the target compound. The effect of the initial dye concentration was also investigated. Moreover, possible dye degradation pathways were proposed based on the determination of possible intermediates using UV–Vis scanning and high-performance liquid chromatography in combination with mass spectrometric (HPLC-MS) detection methods, activity analysis of key enzymes, as well as relevant literatures. In addition, acute toxicity of the selected dye before and after biodegradation was analyzed by the Microtox method to assess the detoxification effectiveness of the pure culture.

### The Influence of SMF on the Strain

Based on the characterization study, the effect of SMF of different intensities (0, 24.6, 41.4, 95.0, 206.3, and 305.0 mT) on the yeast culture was studied through batch experiments. Permanent magnets of different shapes were used for supplying SMF, as described by Tan et al. (2020). The processes of color removal and cell multiplication were monitored and compared to determine the optimal SMF intensity. Then, the influence of SMF with the optimal intensity on the yeast’s halotolerance was further investigated. Key enzyme activity was assessed to investigate the influence of SMF on metabolic capacity.

### Assays

The concentrations of dyes and microbial cells were analyzed using the spectrophotometric method as described by Tan et al. (2013). High-performance liquid chromatography in combination with mass spectrometric analysis was conducted with an Agilent 1260-LC/6130B-MS combined system (Agilent Technologies Inc., CA, United States) under the previously described operation parameters (Tan et al., 2016). Acute toxicity was assessed using the Microtox method (Li H. et al., 2015). The activities of laccase (Lac), manganese peroxidase (MnP), lignin peroxidase (LiP), and nicotinamide adenine dinucleotide-dependent 2,6-dichlorophenolindophenol (NADH-DCIP) reductase were analyzed using the “time-course” procedure of a UV–Vis spectrophotometer according to the reaction system and operation parameters described by a previous literature (Song et al., 2017). Specific activity of NADH-DCIP reductase was defined as 1 μg of DCIP consumed per milligram of protein per minute (Liu et al., 2013). One unit of LiP, MnP, and Lac was defined as 1 μmol of substrate consumed (or product generated) per milliliter of the crude enzyme solution per minute, and specific activity was defined as enzyme units per milligram of protein (Arora and Gill, 2001). The protein concentration was measured according to Bradford (1976). All the analytical tests were performed in triplicate.

### Transcriptome Sequence Analysis

Two groups of yeast cells were incubated under the optimal conditions for 12 h to completely decolorize 80 mg/L ARB in the presence and absence of the optimal-intensity SMF, respectively, corresponding to the experimental group and the control. Then, yeast cells were collected, pretreated, and stored for transcriptome sequencing according to the method by Tan et al. (2020).

#### Transcriptome Sequencing Procedure

RNA isolation and the following preparation of the cDNA library were performed according to Liu et al. (2017). Then, the transcriptome was sequenced by Novogene Bioinformatics Technology Co., Ltd. (Beijing, China).

#### Bioinformatics Analysis

Raw data of the transcriptome sequence was first cleaned and assembled according to the previous research (Li X. et al., 2015) before bioinformatics analysis. Then, gene functions were annotated using databases including the NCBI non-redundant protein sequence (Nr) and non-redundant nucleotide sequence (Nt) databases^1^, Protein family (Pfam)^2^, manually annotated and reviewed section of the UniProt Knowledgebase (SwissProt)^3^, KEGG Ortholog (KO)^4^, and Gene Ontology (GO)^5^. Further bioinformatics analysis including coding sequence (CDS) prediction, gene expression-level analysis, and differentially expressed genes (DEGs) analysis were performed according to Li and Dewey (2011). Differentially expressed genes were identified as the expressed genes with a false discovery rate (FDR) of <0.001 and the reads per kilobase of exon model per million mapped reads (RPKM) ratio of two samples of >2.0 (Audic and Claverie, 1997; Bullard et al., 2010). The fold change of DEGs was presented as the log_2_ fold change (log_2_ FC) of gene abundance, as described by Tan et al. (2020). DEGs with log_2_ FC ≥ 1 or log_2_ FC ≤ -1 were identified as significantly up- or down-regulated, respectively.

#### qRT-PCR Validation

The reliability of the significant DEGs responsible for relevant functions or properties was validated using quantitative real-time PCR (qRT-PCR), which was performed by Sangon Biotech Co., Ltd. (Shanghai, China). Supplementary Table S2 shows the primers for qRT-PCR validation, which were designed based on the sequences of the target genes. Experimental results were calculated and shown according to Schmittgen and Livak (2008) using 18S rRNA gene as the reference.

### Statistical Analysis

One-way analysis of variance (ANOVA) was used for the statistical analysis of experimental data using Origin 2017 software. Data with *P* values that are less than 0.05 are considered as significant.

## Results and Discussion

### Identification and Systematic Characterization of a Newly Isolated Halotolerant Yeast

#### Identification and Azo Dye Decolorization Characteristics

A newly isolated yeast strain, A2, was tested for its ability to decolorize various azo dyes (shown in Supplementary Figure S1A) under hypersaline conditions (30.0 g/L NaCl). Yeast A2 was identified as *Pichia occidentalis* by 26S rDNA sequence analysis (Supplementary Figure S1B). Growing cells of this yeast could decolorize 70.87–99.66% of the six tested azo dyes within 24 h (Supplementary Figure S2). Among the tested dyes, ARB was used for further investigation as the dye was the most rapidly decolorized. To assess the optimal conditions for yeast A2 action, the effects of different parameters were assessed for ARB decolorization and cell multiplication, as shown in Supplementary Figures S3,S4. Within 12 h, ≥ 96.24% (the highest) of 80 mg/L ARB was decolorized under the following conditions: glucose, ≥ 4.0 g/L; (NH_4_)_2_SO_4_, ≥ 1.0 g/L; yeast extract, ≥ 0.1 g/L; NaCl, ≤ 30.0 g/L; rotation speed, ≥160 rpm; temperature, 30°C; and pH, 5.0. Yeast A2 also showed the best growth performance under these conditions. Thus, these were determined as the optimal conditions for yeast A2.

#### Proposed ARB Degradation Pathway

**FIGURE 1:**
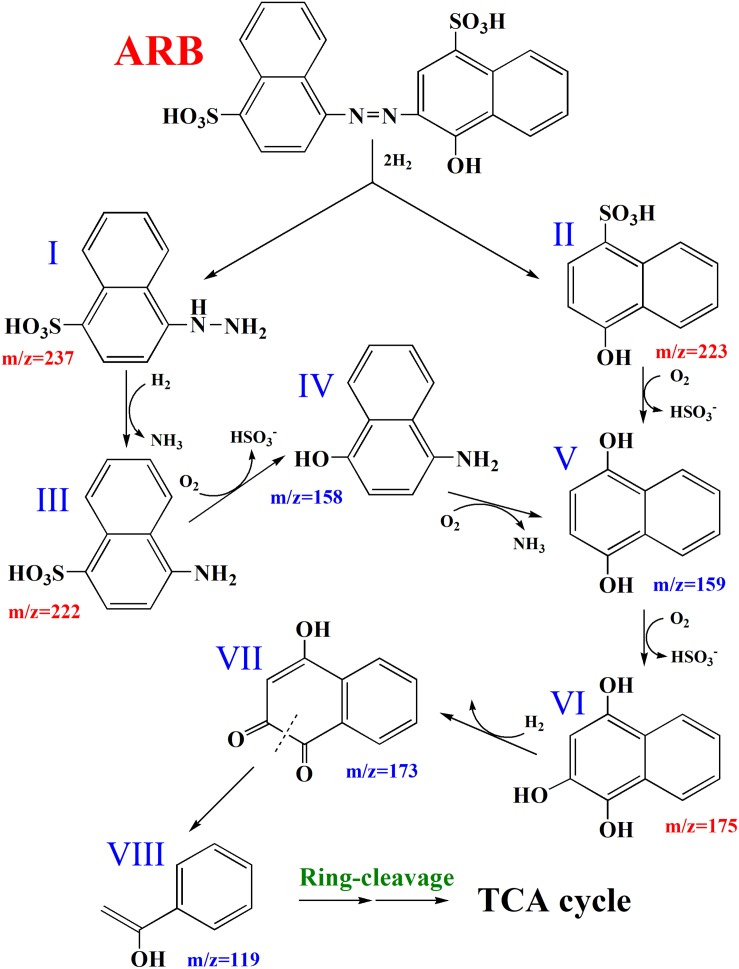
Proposed partial pathways for Acid Red B (*ARB*) degradation by yeast A2. I, 4-hydrazinylnaphthalene-1-sulfonic acid; II, 4-hydroxynaphthalene-1-sulfonic acid; III, 4-aminonaphthalene-1-sulfonic acid; IV, 4-aminonaphthalen-1-ol; V, naphthalene-1,4-diol; VI, naphthalene-1,2,4-triol; VII, 4-hydroxynaphthalene-1,2-dione; VIII, 1-phenylethenol.

Supplementary Figure S1A shows that cell pellets were not colored after the decolorization of ARB, which suggested that ARB might be decolorized by yeast A2 through biodegradation. Additionally, the UV–Vis scanning spectrum of the original ARB solution (50 mg/L) was obviously changed after decolorization, as indicated by the significant decrease or increase of intensity at 516, 323, and 254 nm (as shown in Supplementary Figure S5A). This suggested that the azo group and naphthyl ring were decomposed into aromatics, which were first accumulated during decolorization and then were further degraded over an extended period of time. Based on the HPLC-MS analysis, four possible metabolites were identified: 4-hydrazinylnaphthalene-1-sulfonic acid, 4-hydroxynaphthalene-1-sulfonic acid, 4-aminonaphthalene-1-sulfonic acid, and naphthalene-1,2,4-triol (Supplementary Figure S5B), with *m*/*z* values of 237.0, 223.0, 222.1, and 175.0, respectively (Arun et al., 2013; Adnan et al., 2015). Additionally, the activities of four possible key enzymes involved in dye decolorization were analyzed, as shown in Table 1. The results showed that the intracellular activities of LiP, MnP, and Lac were determined as 0.324 ± 0.00028, 0.390 ± 0.00055, and 0.129 ± 0.000084 U/mg protein, respectively. No activity of NADH-DCIP reductase was detected. Based on these results and previous reports (Song et al., 2017; Tan et al., 2019), a possible degradation pathway of ARB by yeast A2 was proposed and is shown in Figure 1. Azo dyes are generally biodegraded through the cleavage of azo groups, in a process that might rely on some reductases or oxidoreductases (Liu et al., 2013). For this process, the first step was probably catalyzed by Lac, according to the enzymatic analysis result. Previous studies suggested that the cleavage position of the azo group might be the N=N bond or the adjacent C–N bond (Tan et al., 2013, 2019). In this process, the cleavage is likely of the C–N bond, with the detection of intermediates I and II by HPLC-MS analysis. Compound I could be reductively deaminated into compound III (detected), relying on the activity of some reductases. Subsequently, compounds II and III might be further decomposed into compounds IV, V, and VI (detected) through oxidative deamination or desulfurization, processes which might be catalyzed by LiP and/or MnP (Tan et al., 2019). Finally, compound VI could be further metabolized through open-loop of hydroxy-substituted naphthalene and a final tricarboxylic acid (TCA) cycle.

**TABLE 1:** Activities of four key enzymes of yeast A2 with and without 206.3 mT static magnetic field (SMF).

**Key enzymes**	**Without SMF**		**With 206.3 mT SMF**	
	
	**Intracellular**	**Extra cellular**	**Intracellular**	**Extra cellular**
LiP^a^	0.324 ± 0.00028	N.D.	0.323 ± 0.0031	N.D.
MnP^a^	0.390 ± 0.00055	N.D.	0.451 ± 0.0046	N.D.
Lac^a^	0.129 ± 0.000084	N.D.	0.177 ± 0.00024	N.D.
NADH-DCIP reductase^b^	N.D.	N.D.	N.D.	N.D.

Values are the mean of three experiments ± standard error of the mean and are significantly different from the control at *P < 0.001 by one-way ANOVA with Tukey–Kramer comparison test. *N.D.*, not detected. ^*a*^In units per milligram protein. ^*b*^In micrograms of DCIP reduced per minute per milligram protein.

#### Acute Toxicity Assessment

As shown in Supplementary Figure S6, the inhibition ratio (IR) of 80 mg/L ARB against *Vibrio fischeri* was 88.66 ± 0.043% (in the presence of 30.0 g/L NaCl). After decolorization for 12 h, the IR (96.44 ± 0.028%) was higher than that before treatment, indicating the possible accumulation of intermediates with higher acute toxicity. Furthermore, when the treatment time was extended to 24 h, the IR sharply decreased to about 15.64 ± 0.033%, corresponding to a micro-toxicity level. This degradation depended on the non-specific oxidoreductive enzymes (the LiP and MnP shown in Table 1) and resulted in an obvious decrease in the acute toxicity of ARB and its toxic decolorization intermediates.

### SMF-Induced Effects on Yeast A2 for Azo Dye Decolorization and Halotolerance

**FIGURE 2:**
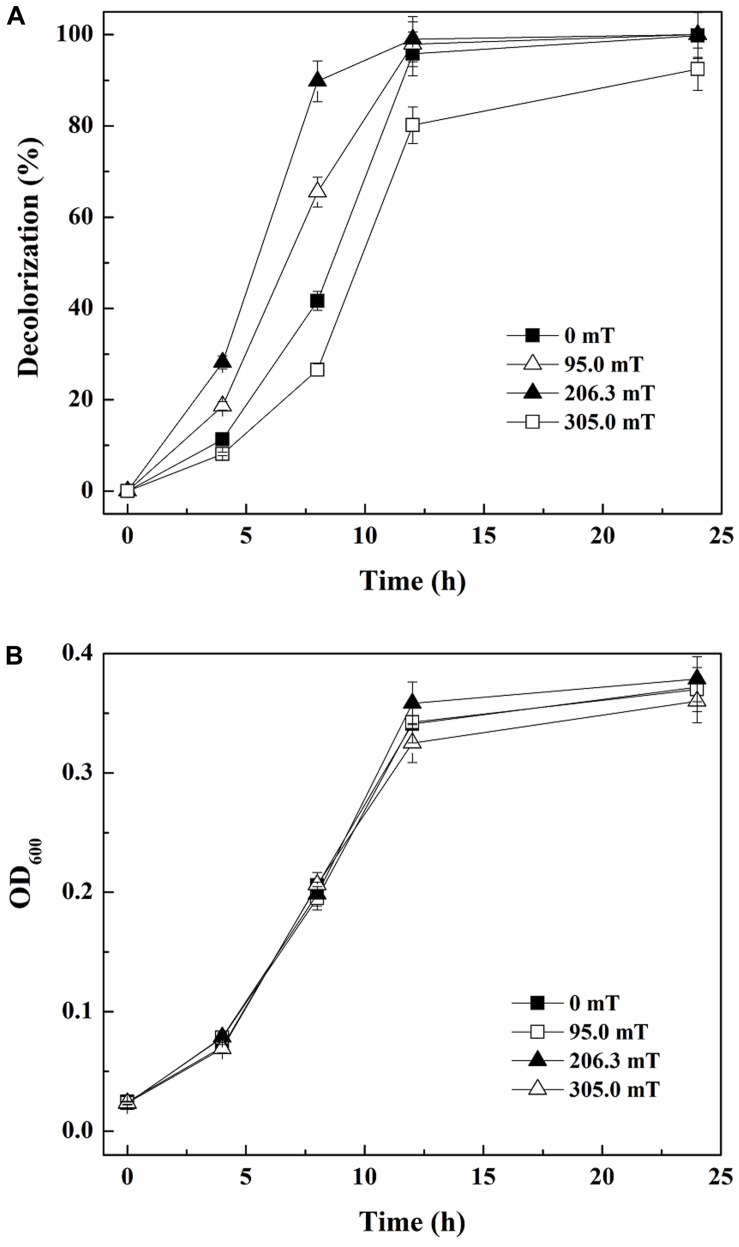
Decolorization of 80 mg/L Acid Red B (*ARB*) by growing cells of yeast A2 **(A)** and cell growth (the OD_600_ values were analyzed after 10-fold dilution) **(B)** with static magnetic field (SMF) of different intensities.

As reported by Miyakoshi (2005), SMF of different intensities can have various effects on microorganisms. Figure 2A demonstrates that 41.67% of 80 mg/L ARB was decolorized within 8 h by yeast A2 in the absence of SMF. When exposed to SMFs of 95.0 and 206.3 mT, the decolorization percentages were more than 65.49 and 87.77%, respectively, both higher than the one without SMF. However, when the time was extended to 12 h, the decolorization percentages with 0, 95.0, and 206.3 mT SMFs approached 100% (95.80–99.00%). In comparison, the 12-h decolorization percentage was 80.15% with exposure to 305.0 mT SMF, which was the lowest among all the groups. This suggested that SMFs of 95.0 and 206.3 mT improved ARB decolorization by yeast A2, but 305.0 mT SMF inhibited the process. Additionally, 95.0, 206.3, and 305.0 mT SMFs had little effect on cell growth compared with the control (without SMF), as determined from the almost coincident growing curves (Figure 2B). Considering both decolorization and cell growth, 206.3 mT was determined as the optimal SMF intensity.

**FIGURE 3:**
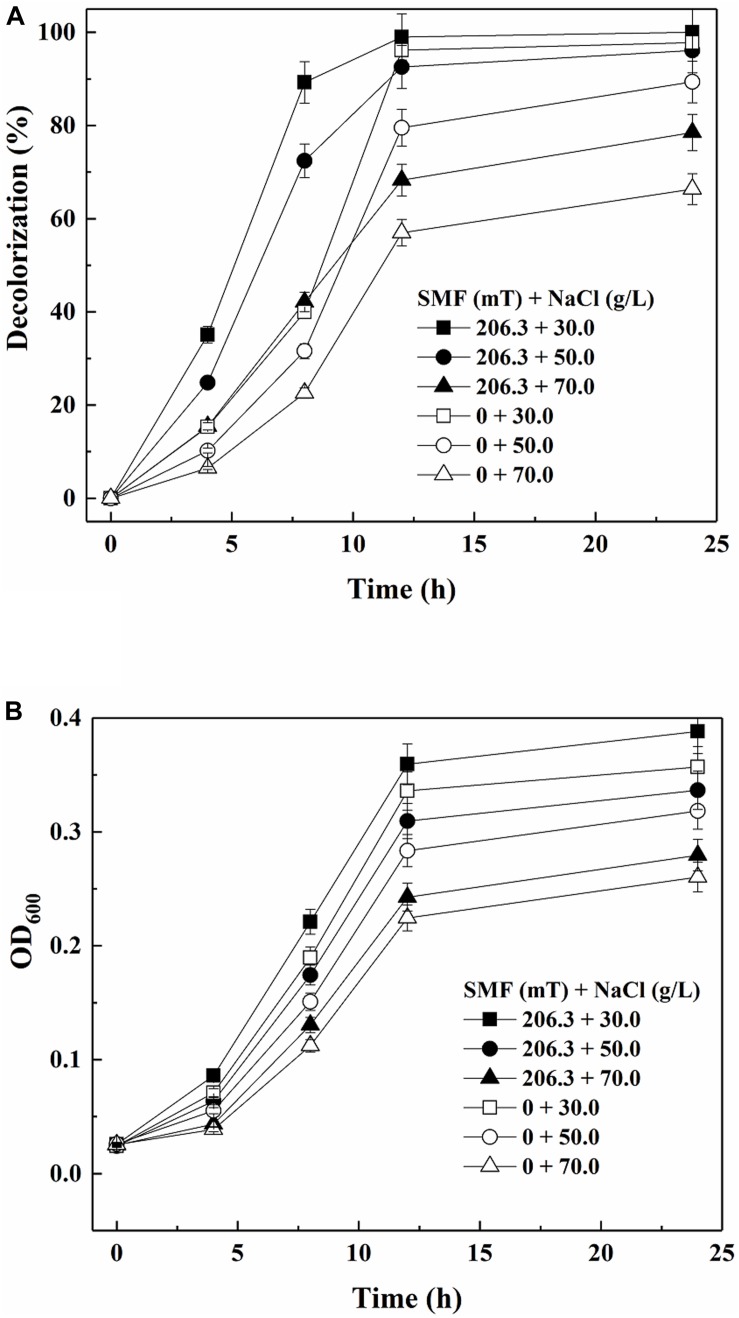
Decolorization of 80 mg/L Acid Red B (ARB) by growing cells of yeast A2 **(A)** and cell growth (the OD_600_ values were analyzed after 10-fold dilution) **(B)** under different salinity conditions with or without the optimal-intensity (206.3 mT) static magnetic field (SMF).

Figure 3A shows the color removal results by yeast A2 in the absence or presence of 206.3 mT SMF under different salinities. As discussed above, the decolorization efficiency was increased by 206.3 mT SMF at a NaCl concentration of 30.0 g/L. When the NaCl concentration was further increased to 50 and 70 g/L, the 12-h decolorization percentages without SMF were 79.50 and 57.00%, respectively. In comparison, the corresponding 12-h decolorization percentages increased to 92.58 and 68.29%, respectively, with exposure to 206.3 mT SMF. As shown in Figure 3B, cell multiplication was also improved by 206.3 mT SMF. Therefore, the results indicated that 206.3 mT SMF enhanced the halotolerance of yeast A2.

### Enzyme Activity Analysis of Yeast A2 With and Without SMF

Based on previous studies, the activities of enzymes putatively involved in azo-degrading processes were determined and compared in the absence and presence of 206.3 mT SMF (Song et al., 2017). As shown in Table 1, enzymatic activities were only determined intracellularly. The activities of two peroxidases (LiP and MnP) and another oxidoreductase (Lac) were detected, but no activity was detected for NADH-DCIP reductase. The LiP activities in the absence and presence of 206.3 mT SMF were 0.324 ± 0.00028 and 0.323 ± 0.0031 U/mg protein, respectively, suggesting that SMF displayed little influence on LiP activity. In addition, the activity of Lac was 1.37-fold elevated (from 0.129 ± 0.000084 to 0.177 ± 0.00024 U/mg protein) by the SMF. The observed increase of Lac activity might be related to the increase of decolorization efficiency because Lac was previously shown to be responsible for azo dye decolorization (Gomi et al., 2011). The activity of MnP with 206.3 mT SMF was 0.451 ± 0.0046 U/mg protein, which was 1.16-fold higher than that without SMF (0.390 ± 0.00055 U/mg protein). MnP might contribute to the further decomposition of toxic intermediates (Kües, 2015); thus, the increased MnP might be related to the increased detoxification effectiveness of yeast A2.

### Possible SMF-Influencing Mechanisms Through Transcriptome Sequencing

#### Gene Function Annotation and Classification

**FIGURE 4:**
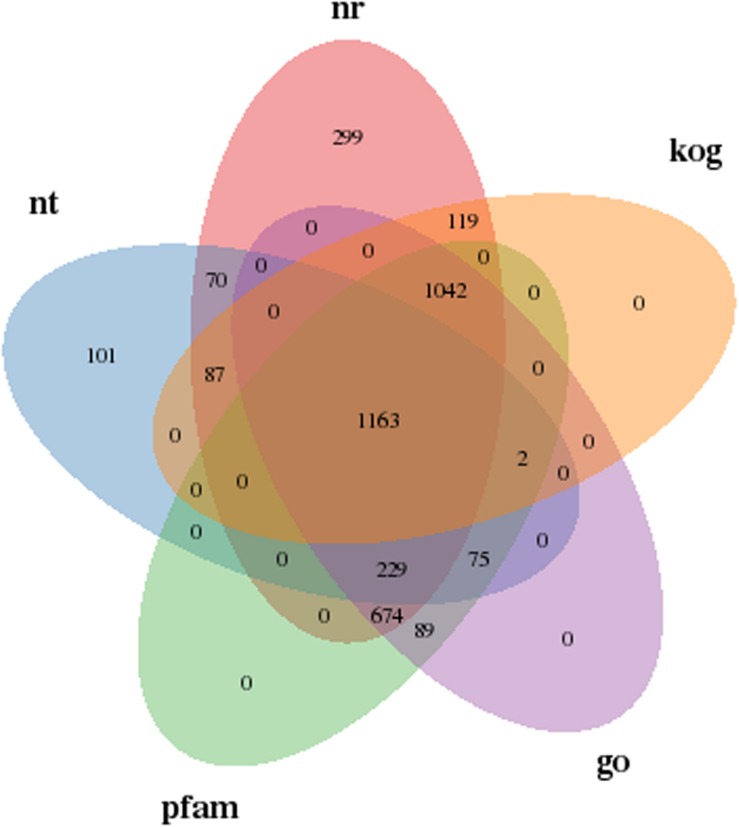
Annotated Venn diagram analysis. Nr, NCBI non-redundant protein sequence database; Nt, NCBI nucleotide sequence database; KOG, euKaryotic Ortholog Groups database; Pfam, Protein family database; GO, Gene Ontology database.

To further understand the influencing mechanisms of SMF on yeast A2, a transcriptome sequencing approach was used to analyze the DEGs between yeast grown in the presence and absence of 206.3 mT SMF. The gene annotation results in Supplementary Table S3 shows that the expression levels of 4,289 genes were successfully compared. Of these, 3,954 and 1,043 genes were annotated in at least one of the seven databases and all of these databases, respectively, accounting for 92.18 and 24.31% of the total. In addition, 1,163 genes were simultaneously compared successfully in the Nt, Nr, KOG, GO, and Pfam databases, as shown in Figure 4. Gene function annotation classification using the GO, KOG, and KEGG databases was performed, as shown in Supplementary Figure S7.

#### Differential Expression Analysis

**FIGURE 5:**
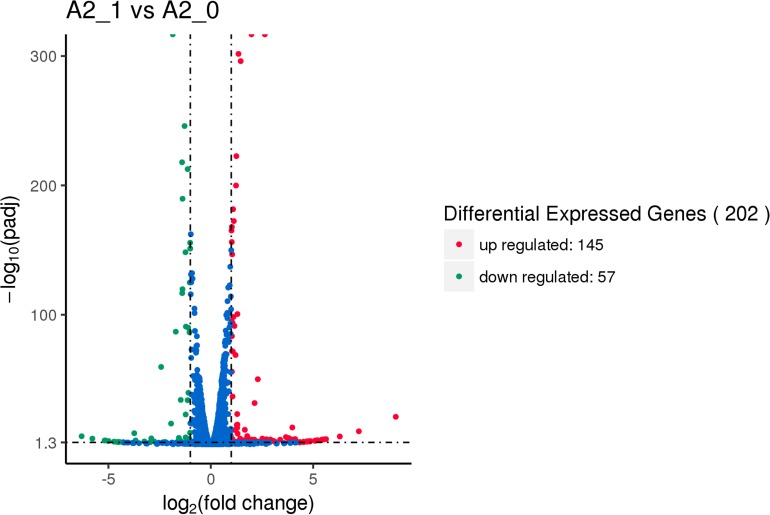
Volcano map of differential expression genes between the experimental group [with 206.3 mT static magnetic field (SMF)] and control (without SMF). Significant differential expression genes are shown as a *red* (up) or *green* (down) *dot*. Non-significant difference between the expressions of genes is shown as a *blue dot*. *Abscissa* represents multiple genes expressed in different samples. *Ordinate* represents the magnitude of gene expression changes.

After being exposed to 206.3 mT SMF, there were 202 (145 up- and 57 downregulated) significant DEGs of yeast A2 compared to the SMF-absent control (shown in Figure 5). The above results suggested that 206.3 mT SMF improved ARB decolorization performance and halotolerance of the yeast, but had little effect on cell multiplication. Thus, the DEGs which might be putatively involved in biodegradation and halotolerance were further analyzed.

The expression levels of genes encoding enzymes putatively involved in the biodegradation of ARB and possible decolorization intermediates were determined in the transcriptome analysis (shown in Table 2). Lac was reported to be involved in azo dye degradation and belongs to a family of multicopper oxidases (Jones and Solomon, 2015). One gene (K19791/PF00394) encoding multicopper oxidase was determined as not significantly upregulated (the log_2_ FC was 0.40). This result might be consistent with the Lac activity being 1.37-fold higher after being exposed to SMF. In addition, the levels of four genes encoding NADH-ubiquinone oxidoreductase were measured, as this enzyme was previously suggested to be involved in azo dye decolorization (Mahmood et al., 2017). Of these four genes, two genes (O47950 and Q02854) were upregulated (indicated by log_2_ FC of 0.32 and 0.38) and the other two genes (PF07347 and PF10588) were downregulated (log_2_ FC of -0.32 and -0.31). This might also be consistent with the slight increase in decolorization efficiency. One gene (K00428) encoding cytochrome c peroxidase (putative MnP) was also determined as slightly upregulated (log_2_ FC was 0.19) in the transcriptome, which was consistent with the 1.16-fold elevation of MnP activity observed with SMF. Overall, although genes putatively involved in dye decolorization and further degradation of intermediates were identified, none showed significant changes in expression. Therefore, the dye decolorization efficiency with 206.3 mT SMF was only slightly higher than that without SMF.

**TABLE 2:** Relative expression levels of the selected genes by transcriptome sequencing and qRT-PCR validation (only for significantly up- or downregulated genes).

**Description**	**Gene ID**	**Enzyme**	**Transcriptome (log_2_ FC)^a^**	**Adjusted *P*-value^b^**	**qRT-PCR validation (relative expression fold)**
Halotolerance	O59841	Glyceraldehyde-3-phosphate dehydrogenase	1.10	5.23E-271	1.63
	P39932	Sugar transporter STL1	1.01	1.86E-266	1.39
	Q5A5U4	Cell wall protein RHD3	1.00	2.97E-88	1.04
Dye degradation	K00428	Cytochrome c peroxidase	0.19	1.99E-8	None
	K19791/PF00394	Multicopper oxidase	0.40	3.09E-19	None
	PF07731	Multicopper oxidase	–0.03	0.6345	None
	O47950	NADH-ubiquinone oxidoreductase, 20-kDa subunit	0.32	2.09E-20	None
	Q02854	NADH-ubiquinone oxidoreductase, 20.9-kDa subunit	0.38	9.71E-25	None
	PF07347	NADH-ubiquinone oxidoreductase subunit B14.5a	–0.32	8.00E-21	None
	PF10588	NADH-ubiquinone oxidoreductase-G iron–sulfur binding region	–0.31	8.32E-7	None

*^*a*^Log_2_ FC > 1 represents upregulation; log_2_ FC < -1 represents downregulation; log_2_ FC range from -1.0 to 1.0 represents insignificant regulation. ^*b*^Adjusted *P* value < 0.05 represents significance; otherwise, represents insignificance.*

The results shown in Figure 2 demonstrated that 206.3 mT SMF enhanced the halotolerance of yeast A2. Accordingly, three significantly upregulated genes encoding proteins (annotated in the SwissProt database) putatively involved in hyperosmolality resistance were determined and the expression levels were validated (shown in Table 2). One gene (P39932) encoding putative glycerol proton symporter Stl1 was 1.01-fold upregulated by 206.3 mT SMF under hypersaline conditions (30.0 g/L NaCl). As described by Hohmann (2002) and Zemancíková et al. (2018), glycerol proton symporters (Stl1 and Stl2) are important for the osmotic stress resistance of yeasts (e.g., *Dekkera bruxellensis* and *Saccharomyces cerevisiae*) through glycerol synthesis and its intracellular accumulation. Most prokaryotic and eukaryotic organisms tolerate hypersaline conditions using an “organic solute-in strategy” through the accumulation of “compatible solutes” (e.g., polyols in fungi) to maintain their intracellular Na^+^ concentrations below the levels that would be toxic for the cells (Hohmann, 2002). *Debaryomyces hansenii* and *S. cerevisiae* accumulated glycerol intracellularly as their main compatible solute (Jovall et al., 1990). In addition to the intracellular accumulation of glycerol, fungi could also acquire the compatible solutes from the environment when being exposed to high osmotic conditions depending on specific transporters, which exist in the plasma membrane (Hohmann, 2002). This mechanism had been confirmed as being responsible for osmotolerance in yeasts, including *S. cerevisiae*, *Candida albicans*, and *Zygosaccharomyces rouxii* (Ferreira et al., 2005; Kayingo et al., 2009; Duskova et al., 2015). Thus, the upregulation of gene P39932 might be responsible for the elevated halotolerance of yeast A2. In addition, another gene (O59841) encoding glyceraldehyde-3-phosphate dehydrogenase (GPD; with a protein sequence similarity of 83.23%) was 1.10-fold upregulated, which was also putatively related to the elevated halotolerance according to Jeong et al. (2000). Overexpression of the GPD isolated from *Pleurotus sajor-caju* enhanced halotolerance in rice (Cho et al., 2014). Additionally, GPD also participated in glycolysis and glucogenesis in *S. cerevisiae* and *Aspergillus nidulans* (Punt et al., 1990). Glyceraldehyde-3-phosphate could be oxidized to 1,3-bisphosphate glyceric acid by GPD. Additional energy required for cellular adjustment to high osmotic environments was produced during this process in the salt-tolerant cultures (Redkar et al., 1996). Therefore, upregulation of the gene O59841 encoding GPD might be responsible for the increased halotolerance of yeast A2. Hypo-osmotic stress could be countered by the microbial cell wall (Lu et al., 2016). One gene (Q5A5U4) encoding cell wall protein PGA30 was 1.00-fold upregulated. It was indicated that the yeast cell wall could protect cells from osmotic shock and mechanical stresses and helped to establish and maintain cell morphology and structural integrity (Li et al., 2014). Thus, the significant upregulation of Q5A5U4 might also be responsible for the increased halotolerance of yeast A2. Overall, 206.3 mT SMF induced significant upregulation of putative genes which were responsible for the intracellular synthesis (sometimes also accumulation) of glycerol (an important compatible solute) and the regulation of cell wall components, thus enhancing the halotolerance of yeast A2.

## Conclusion

A halotolerant yeast, *Pichia occidentalis* A2, that could decolorize various azo dyes in a high osmotic environment was characterized. Yeast A2 showed optimal azo dye decolorization and cell growth under the following conditions: glucose, 4.0 g/L; (NH_4_)_2_SO_4_, 1.0 g/L; yeast extract, 0.1 g/L; NaCl, 30.0 g/L; rotation speed, 160 rpm; temperature, 30°C; and pH 5.0. ARB was decolorized through being degraded and even detoxified by yeast A2 through a possible pathway including cleavage of the naphthalene-amidine bond, reductive deamination, oxidative deamination/desulfurization, open-loop of hydroxy-substituted naphthalene, and TCA cycle. Under these conditions, 206.3 mT SMF enhanced yeast A2 for dye decolorization and its cell multiplication. The activities of the involved enzymes were also increased by SMF. It was suggested through transcriptomic analysis that the improved halotolerance of the yeast was related to the increase of intracellular synthesis and accumulation of glycerol (an important compatible solute) and the regulation of cell wall components by 206.3 mT SMF.

## Data Availability Statement

The datasets generated for this study can be found in the Pichia occidentalis strain A2 (accession number MK409682).

## Author Contributions

XW and LT designed the experiments and wrote the manuscript. XW and YW carried out the experiments. XW and SN analyzed the experimental results. XW and SS analyzed the sequencing data and developed analysis tools. SN assisted with Illumina sequencing.

## Conflict of Interest

The authors declare that the research was conducted in the absence of any commercial or financial relationships that could be construed as a potential conflict of interest.

## Footnotes

1 ftp://ftp.ncbi.nih.gov/blast/db/

2 http://pfam.xfam.org/

3 http://www.ebi.ac.uk/uniprot/

4 http://www.genome.jp/kegg/

5 http://www.geneontology.org/
