# Supplementary material for: Improving Azo Dye Decolorization Performance and Halotolerance of Pichia occidentalis A2 by Static Magnetic Field and Possible Mechanisms Through Comparative Transcriptome Analysis
Source: Front Microbiol. 2020 May 5;11:712. doi: 10.3389/fmicb.2020.00712 (PMC7216737; doi:10.3389/fmicb.2020.00712)
Supplement: Supplementary file 1 [file Data_Sheet_1.doc]

***Supplementary Material***

**1 TABLES**

**Table S1 Azo dyes used in the present study.**

| **Name** | **Chemical structure** | **Characteristic absorption wavelength (nm)** |
| --- | --- | --- |
| Acid Red B |  | 516 |
| Acid Brilliant Scarlet GR |  | 511 |
| Reactive Brilliant Red K-2G |  | 509 |
| Reactive Yellow 3RS |  | 540 |
| Reactive Brilliant Red X-3B |  | 538 |
| Reactive Green KE-4BD |  | 627 |

**Table S2 Target genes and their sequences.**

| **Gene ID**  **(Swiss-Prot description)** | **Genetic sequences** |
| --- | --- |
| Cluster-295.335  (Cell wall protein RHD3) | TTCTTTCTAATTAAATTAACAACCTTCATCCACACTCGTTTGCACACAAAACTCATTAATATATCTAAAATGCAAATCAAGAAAACCTTACTCGCCACTGCCACTGCTGCAGCTGCTGCAAATGCTCAAACTGTCAACTTGGTGGTCAAATCAGGTAGCAAGGAAATCGACGGCAACACCATTGGCTTCAAGCACGAGGGAGCGGGCATGGACTACGCTTTTCTAGGTACATCGGCACAGTCACAACTCCTAGACTACGATGCCACTTCGAAATCACTTACCTACAATGCGCAATACCCATACTCGTTCAACGTTGACAAGTATGTGTCACTCACTGTTGGTGGTTCCAACTCGGAGATCACCTTCAACAGCGAAAACACTCTTTTGGTGAATGGTACCTCGAGTGGTTTTTACGCATGTAAAAACACCAATGACCCTTACCAATATTCCAAATATTCTTATGAATTGATGTACTACAAGACCGAAGCTCCTCTCGATTGTATTGCATTGAACCTCGCAAACGGACAAAACAGCACCTCTCCAAATTCAACGACATCTTCGTCATTATCCTCCTACGAAGACTCTGGTGCAATGCTTGCACCGGCTGGCGCATTCGCAGTTCTTTTTGGGGCTGCTGCTGCTCTGATCTAA |
| Cluster-295.1508  (Glyceraldehyde-3-phosphate dehydrogenase) | GCCTCATGTACCACCAACTGTTTGGCCCCATTGGCCAAGGCTGTCAACGACAAGTTCGGTATTGAGGCCGGTTTGATGACCACTATCCACTCCATCACCGCCACCCAAAAGACCGTCGATGGTCCATCCCACAAGGACTGGAGAGGTGGTAGAACCGCTTCCGGTAACATCATCCCATCCTCCACCGGTGCTGCCAAGGCTGTCGGTAAGGTCTTACCTGTCTTGGCCGGTAAGTTGACTGGTATGTCCATGAGAGTTCCAACCGTCGATGTTTCCGTTGTTGACTTGACCGTCAACTTGAAGACCCCAACCACCTACGAAGAAATCTGTGCTGCTATGAAGGAAGCATCCGAAGGTGAATTGAAGGGCATTCTCGGTTACACCGAAGATGCTGTTGTCTCCACCGATTTCATCTCCGACTCTAGATCCTCCATCTTCGACGCCAAGGCTGGTATCTTGTTGACTCCAACCTTCGTCAAGTTGATCTCTTGGTACGATAACGAGTACGXX |
| Cluster-295.1385  (Sugar transporter STL1) | TATACATATTCAATCATGTCTTTGGATGAATCAGGATCAAACGCTAACAATTCATTCTTTGAAGACGACAAACTTGATGATGCTTCCCAAAAAGCAGCTAATAACAACATGAAGGATGACGAAAAAAACACCCCAGCTTTACCATTTCTACAGGAAGATGCCATTCCACTCACAAAGTACTCACTGAAATCTTCTACCGCAACTCTGGGTTTAAAAGGTAAAAAGTTGCAACTCTTGATCACCGCTTGTTGTATTATCGGATTTTCACTTTTCGGTTATGACCAAGGTTTGATGGCAGGTATCATTACCGCCCCTCAATTCATTGAAACATTTCCTCCTGTCGGAGGTGGTACTTACCATGCAACCATTGTTCAAGGTGCTGTCACCTCATGTTATGAGTTGGGTTGTTTCTTTGGTGCTCTCTCGGCTATGTACTTGGGTTCCAGACTGGGTAGAAAACTATTAATTATTATTGGTTCAAGTATTATTATCATCGGTACTTTCATTTCGATTTTCCCATTCAAAGGCCATTGGGCATTGGGTCATTTTGTCATTGCTAGAGTTGTGACTGGTTATGGTAATGGTTTCAATACCGCAACCATCCCAATCTACCAATCCGAATTGTCAGAAGCTAAAAATAGAGGTAGATTGGTGAATTTGGAAGGTTCTTTTGTTGCTGTCGGTACCTTGATTGCATACTGGATTGACTTTGGTCTATCTTATGCTTCTGGCTCGGTCTCTTGGAGATTACCTATTTCATTACAAATCATCTTTGCCGGTTTTGTTCTCATTTTCATGATTGGTTTACCAGAATCCCCAAGATTTTTGATTACTCAAAAACGTCTGGATGAAGCTCGTTATGTTTTAGCTCAGCTTCATGATGTTTCTATCAATGATCCAGATTTAATTGAAGAAGTGGTTATTGTTAGAGATGCAAATAACAGATTCGAAAAACAAGTCTCAATCAAGGAACTTTTCAAGCCTTCAAAACACCAATACTTGAGAAGAATGATCATCGGTTCTTCAGGTCAATTTTTCCAACAATTCACTGGTTGTAATGCTGCAATTTACTACTCTACCGTTTTATTTGAAGATACTGTCGGATTAAGCAGAAGATTATCTTTGGTTTTAGGTGGTGTCTTTGCAACTGTTTATGCTATATTCACAATTCCTTCGTTCTTTTTGGTTGACAGACTGGGTAGAAGACCTCTATTCTTAATTGGTGCTTTTGGTCAAGGTATTTCGTTTTTGATTGCATTTGGTTGTTTGACACATAACAATAAGCAAACTGCAAAGGGTGCTGCATTTGGTTTATTCTTGTTTATTGTTTTCTTTGCCTTCACATTGCTACCAATGCCATGGGTCTATCCACCAGAAATCAATTCCATGAGAACAAGAACAATGGCAACTTCAGTATCCACATGTACCAACTGGTTATCTAACTTTGCGGTTGTTATGTTTACACCAATCTTCATTAACCAATCCAAGTGGGGTTGTTACTTATTCTTTGCCATCATGAACTTCCTCTACATTCCAATTATTTACTTCTTCTATCCTGAAACAGCTGGTAGAACATTGGAAGAGATCGATATCATCTTTGCAAAGTCATTTGTTGAAAACACTCCAGCTTATAAGGTTGCAAACGAATTGCCTAAATTAACAAATAAAGAAATCGAAGAAGAAGGAATCCGTCTTGGTCTATACGAAGAAGACGAAAAGGATTTTGGAGTTTACGAACATCGTCCACCTACTCAAAGAGAACTCTCTTACCGTAGTACTGCATTTGAATATCAAATCTAG |

**Table S3 Success rate statistics for gene annotation**.

| **Database for annotation** | **Number of unigenes** | **Percentage (%)** |
| --- | --- | --- |
| Nr | 3683 | 85.87 |
| Nt | 1727 | 40.26 |
| Pfam | 3274 | 76.33 |
| KOG | 2413 | 56.26 |
| SwissProt | 3366 | 78.47 |
| KO | 2185 | 50.94 |
| GO | 3274 | 76.33 |
| All of above | 1043 | 24.31 |
| At least one of above | 3954 | 92.18 |
| **Total unigenes** | **4289** | **100.00** |

**2 FIGURES**


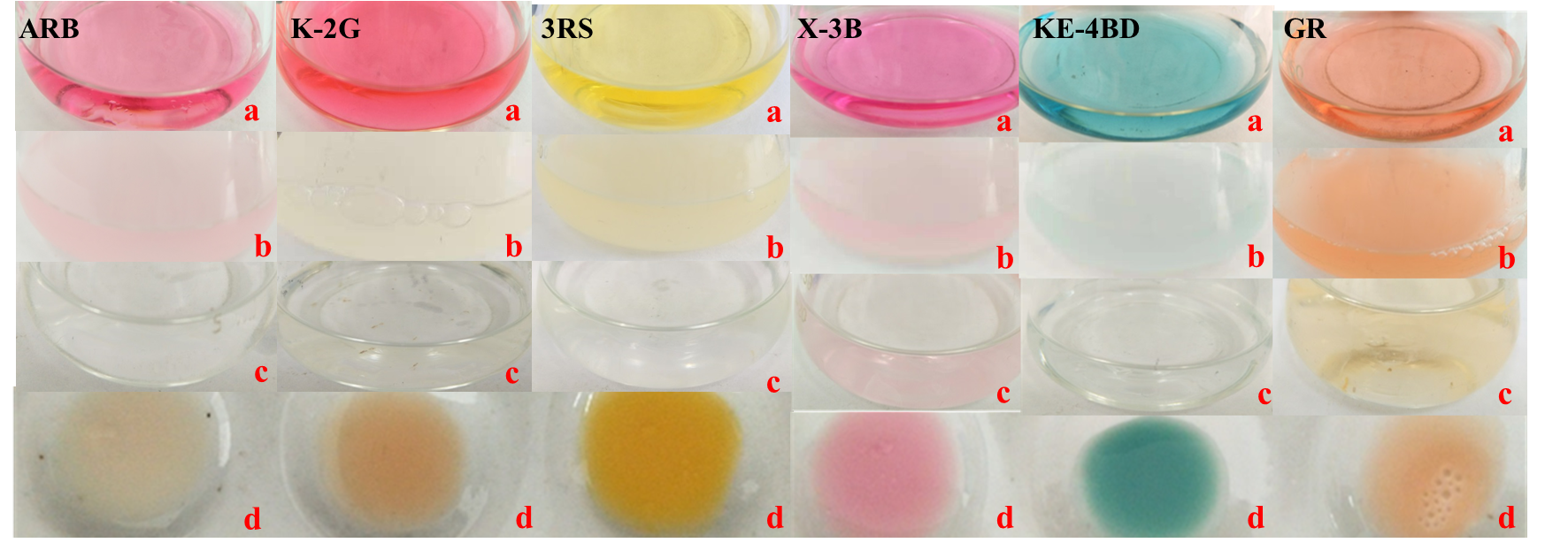


**(A)**


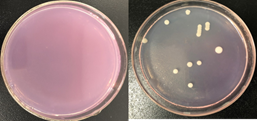

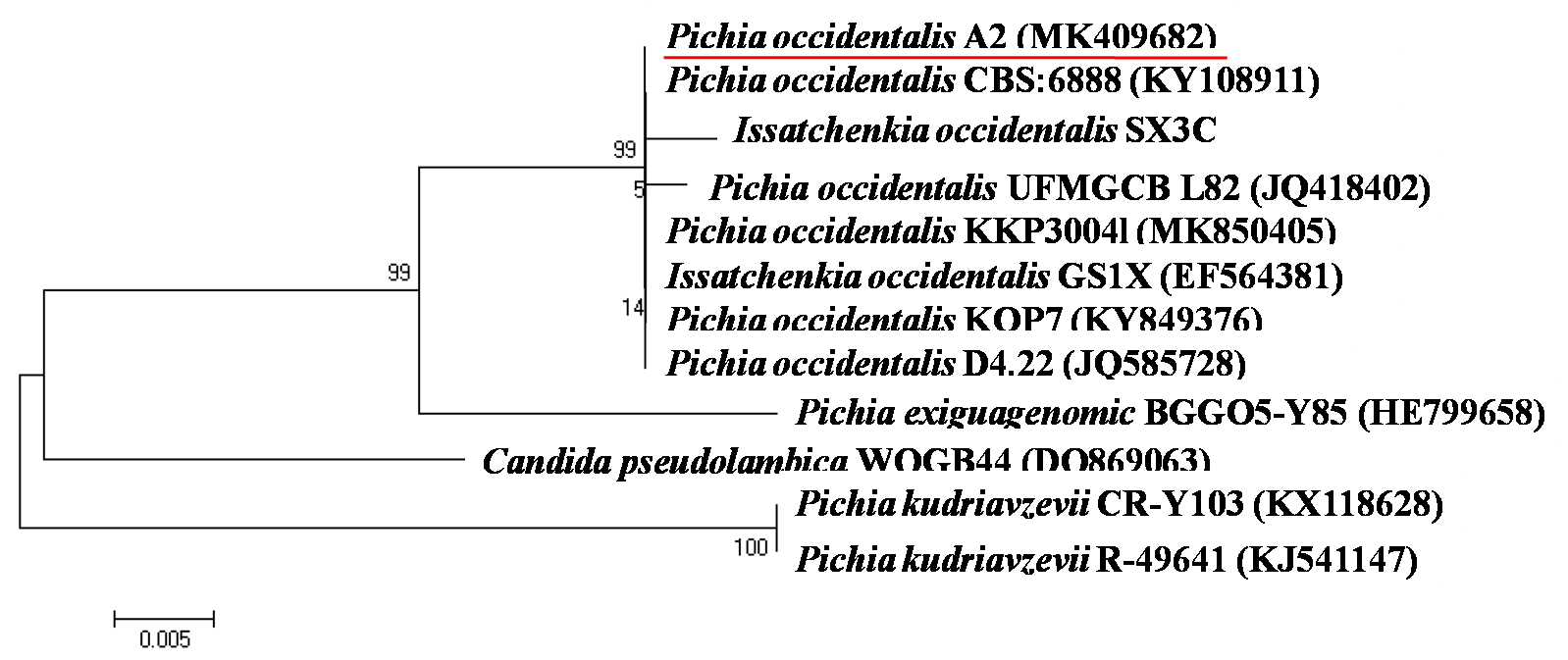


**(B)**

**Fig. S1** A, Photos of (a) original 20 m/L dye solution, (b) cell suspension, (c) supernatant and (d) cell pellets of yeast A2 after decolorization. B, Phylogenetic tree of *Pichia occidentalis* A2 and related species. The GenBank accession number corresponded to each microorganism was exhibited in parentheses. Bootstrap values were showed at each branch. Scale bar indicated 0.005 Jukes-cantor distances.

**
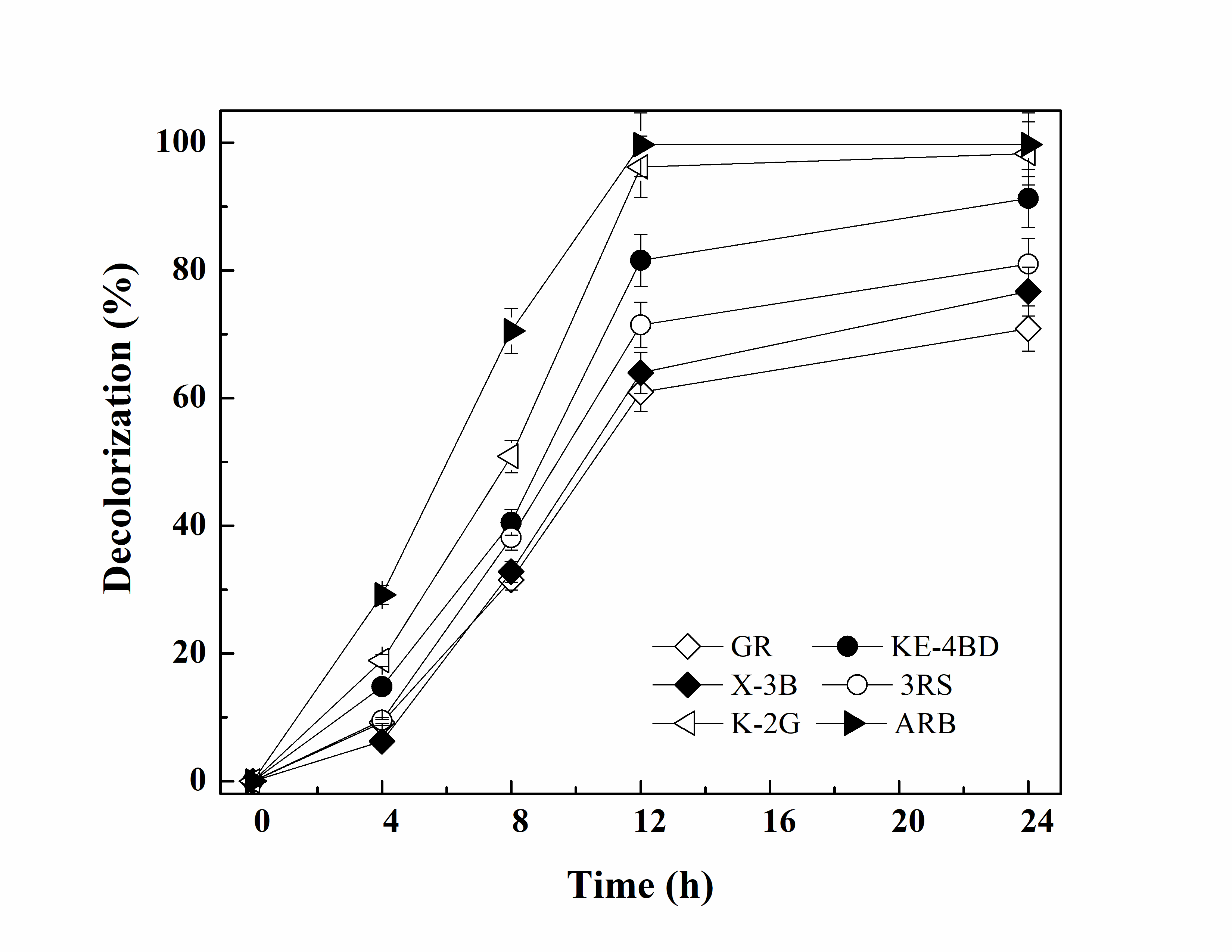

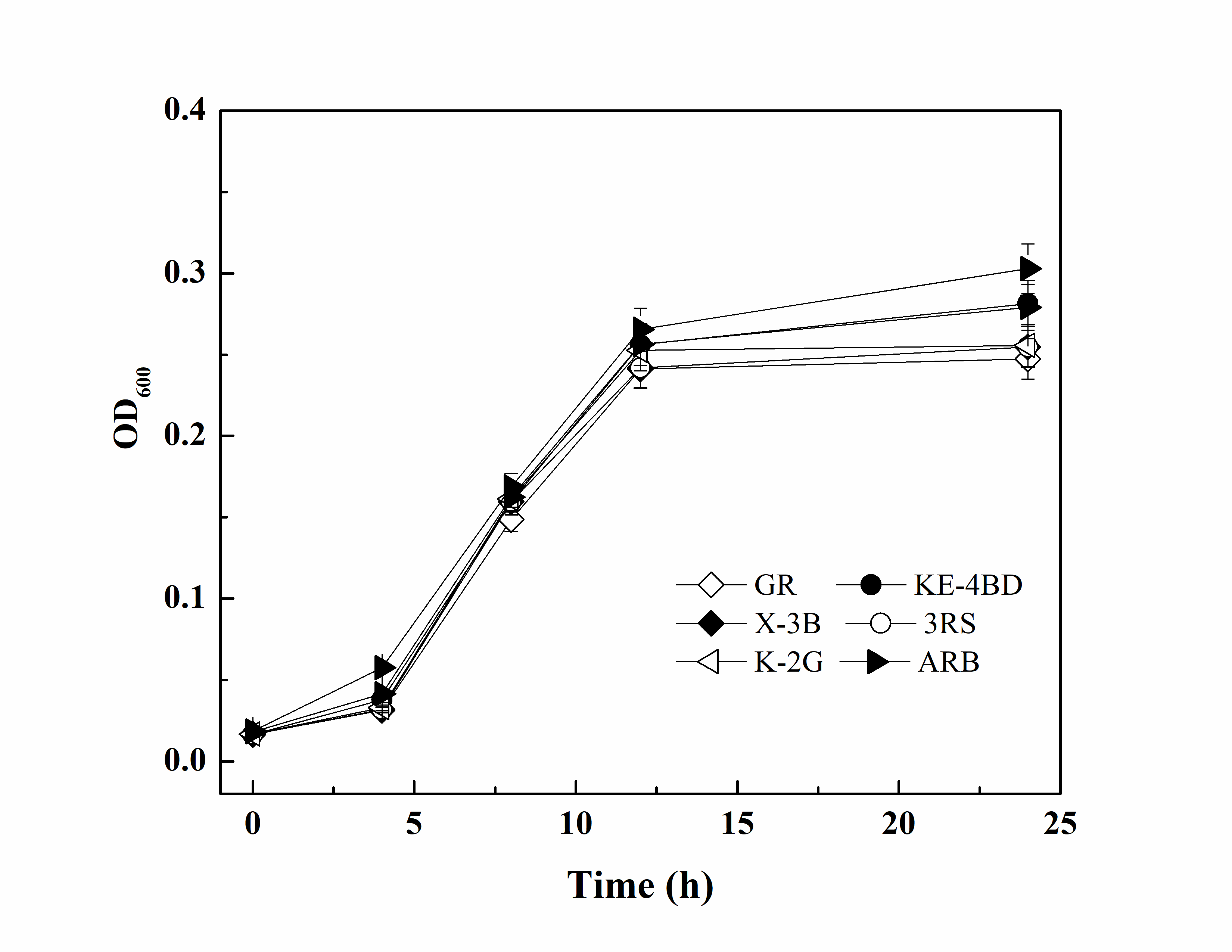
**

**(A) (B)**

**Fig. S2** Decolorization of six different azo dyes (20 mg/L) by growing cells of yeast A2. A, decolorization; B, cell growth (the OD_600_ values were analyzed after 10-fold dilution).


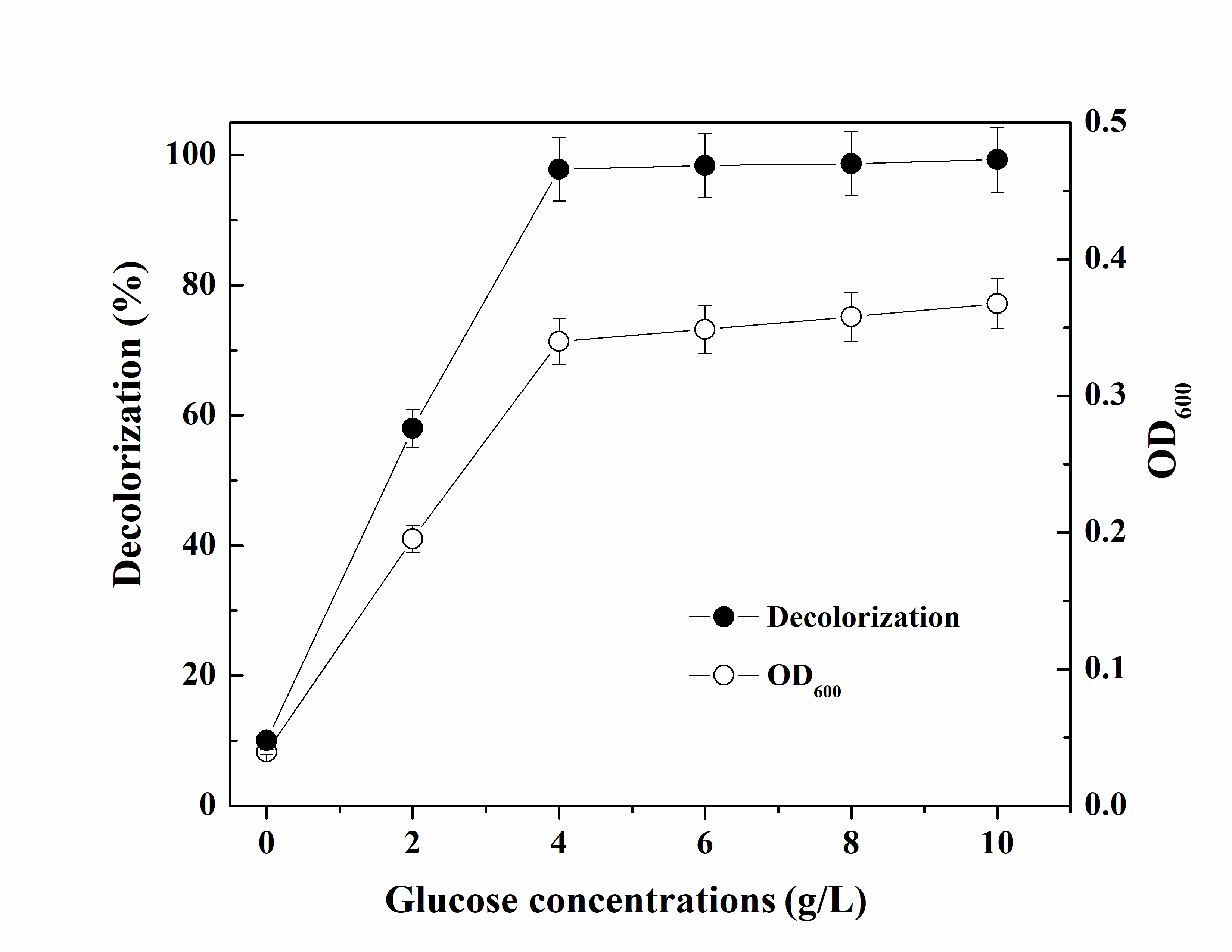

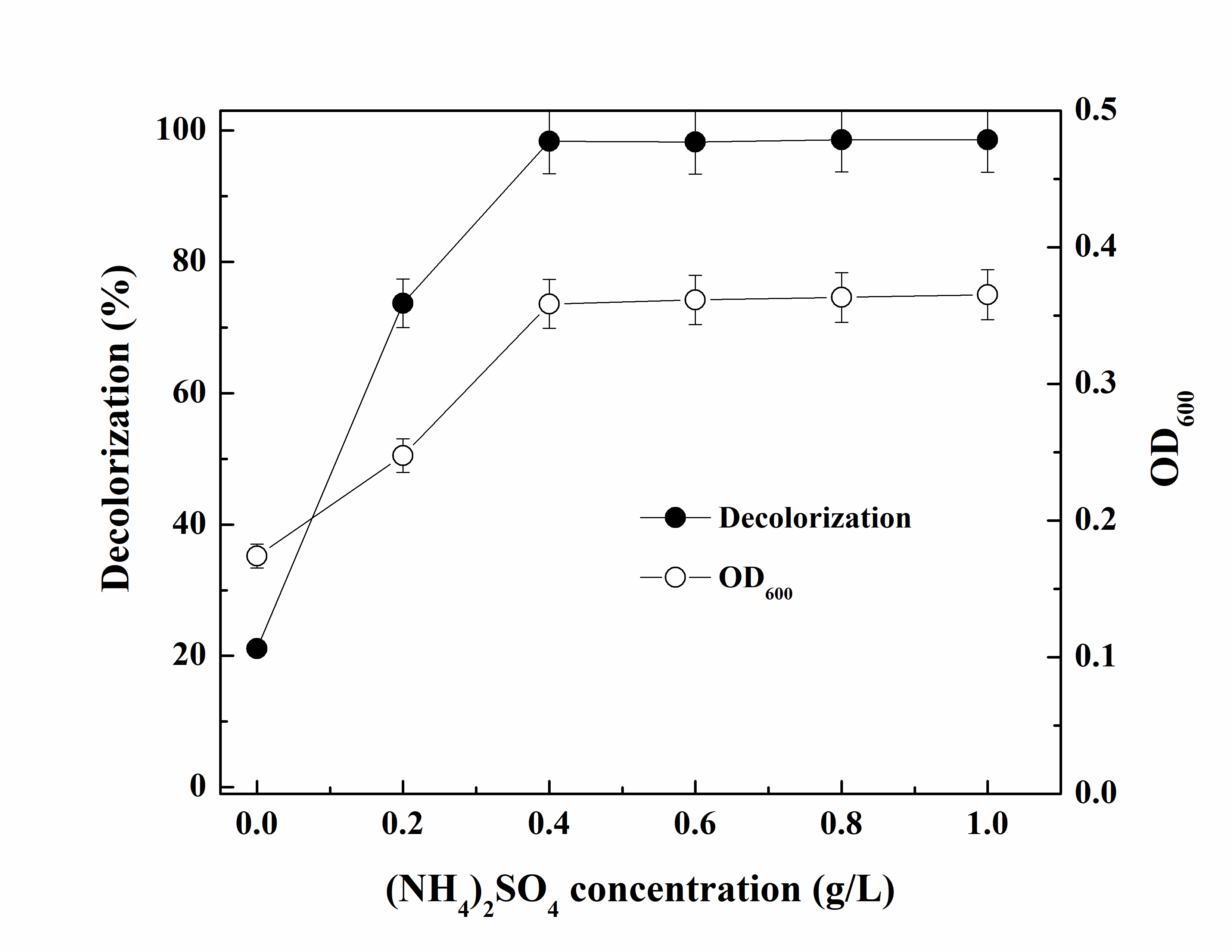


**(A) (B)**


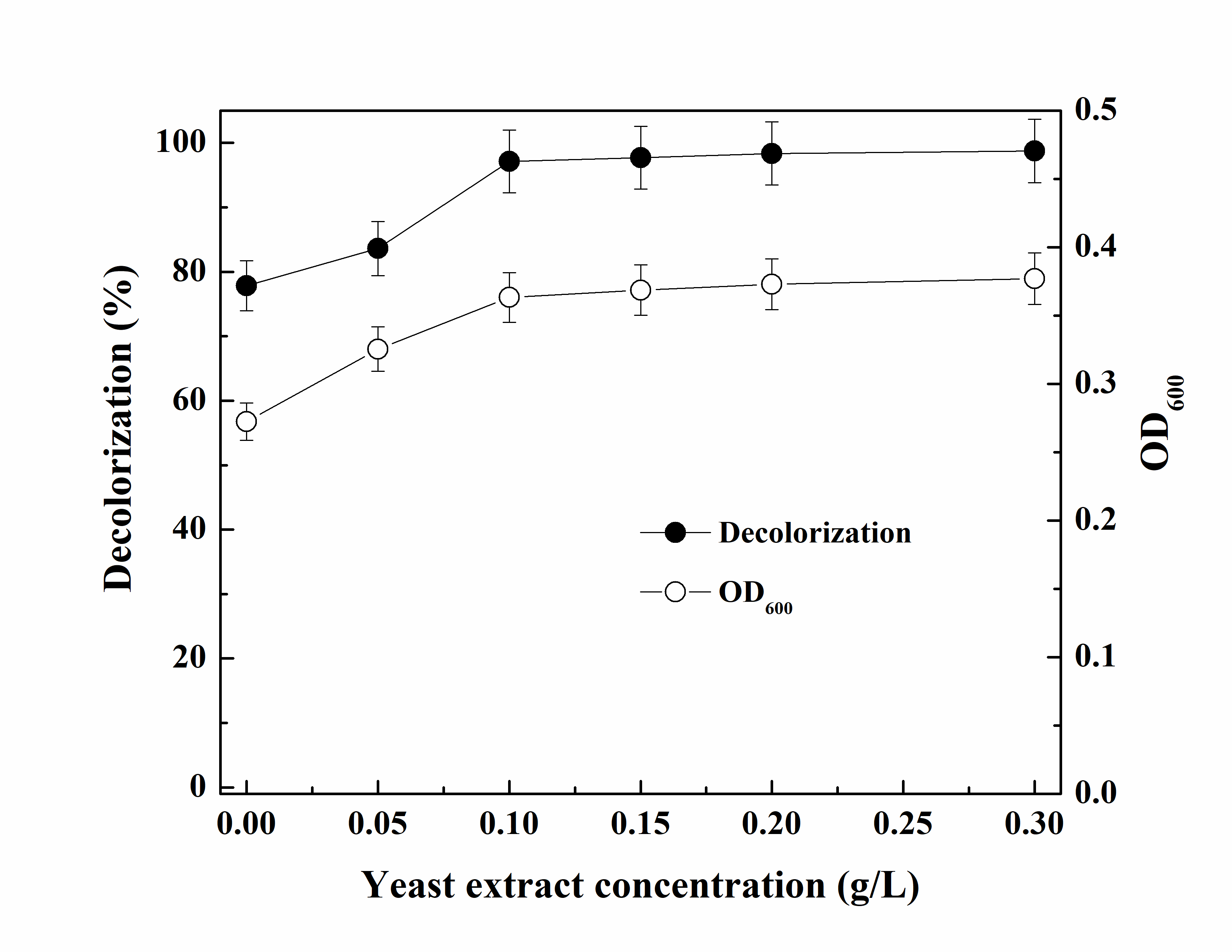

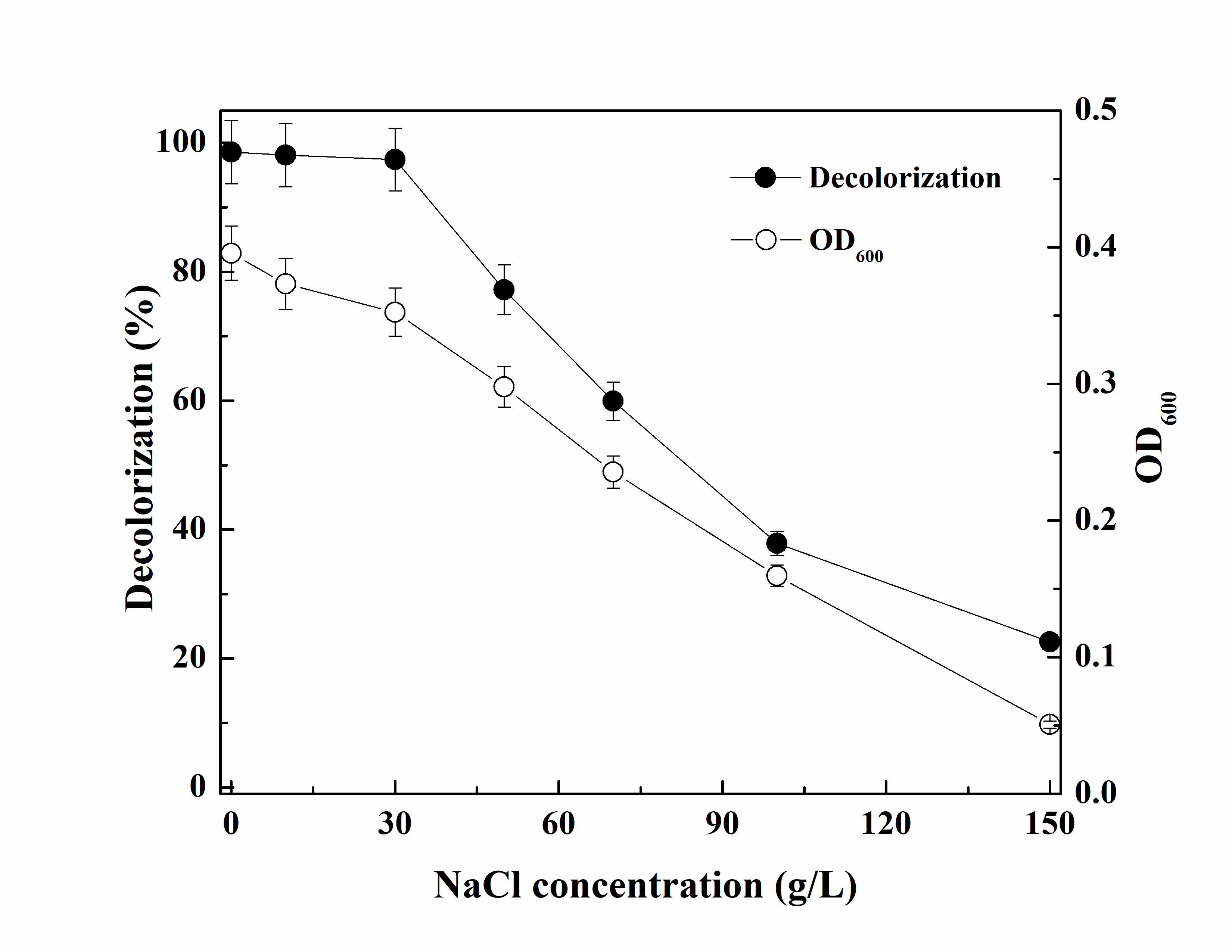


**(C) (D)**

**
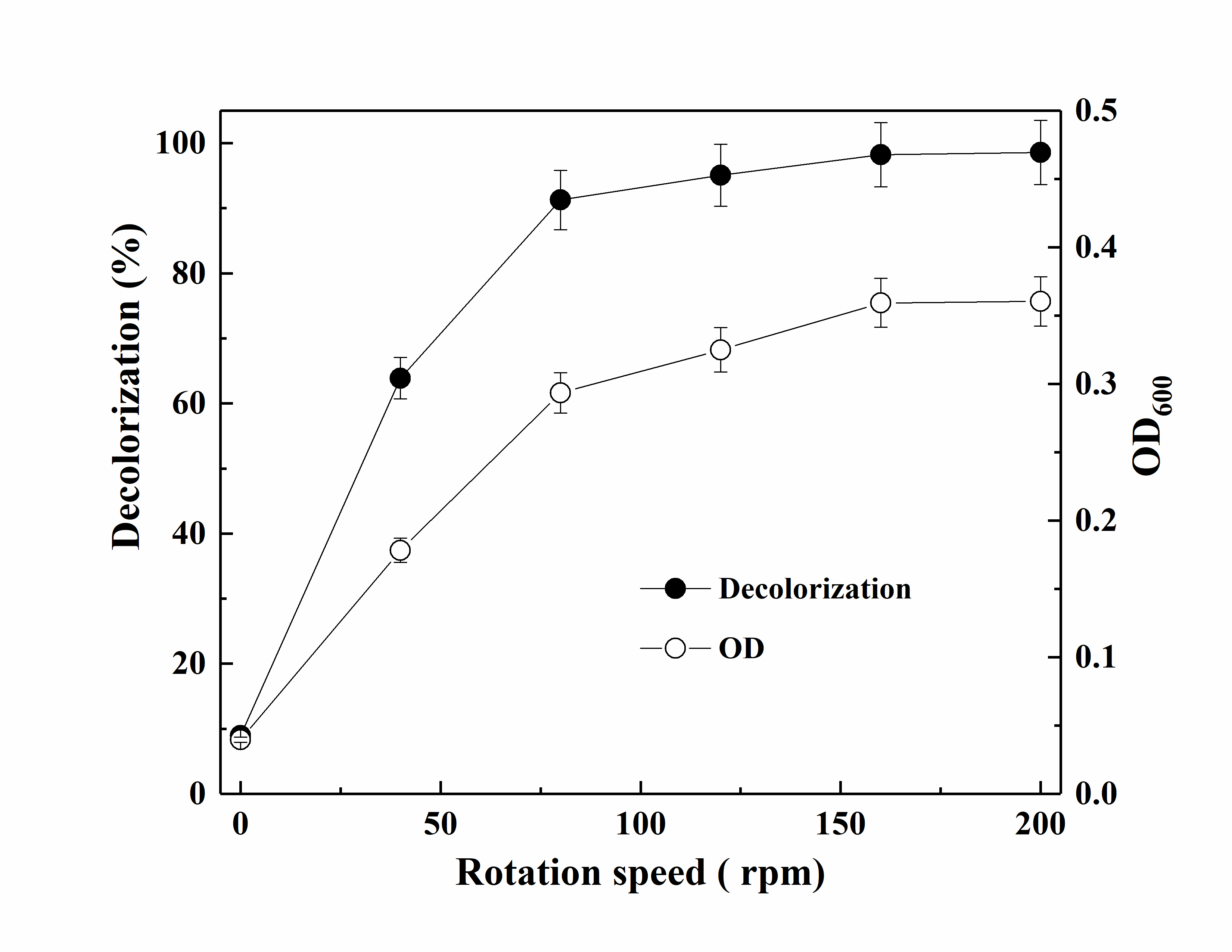

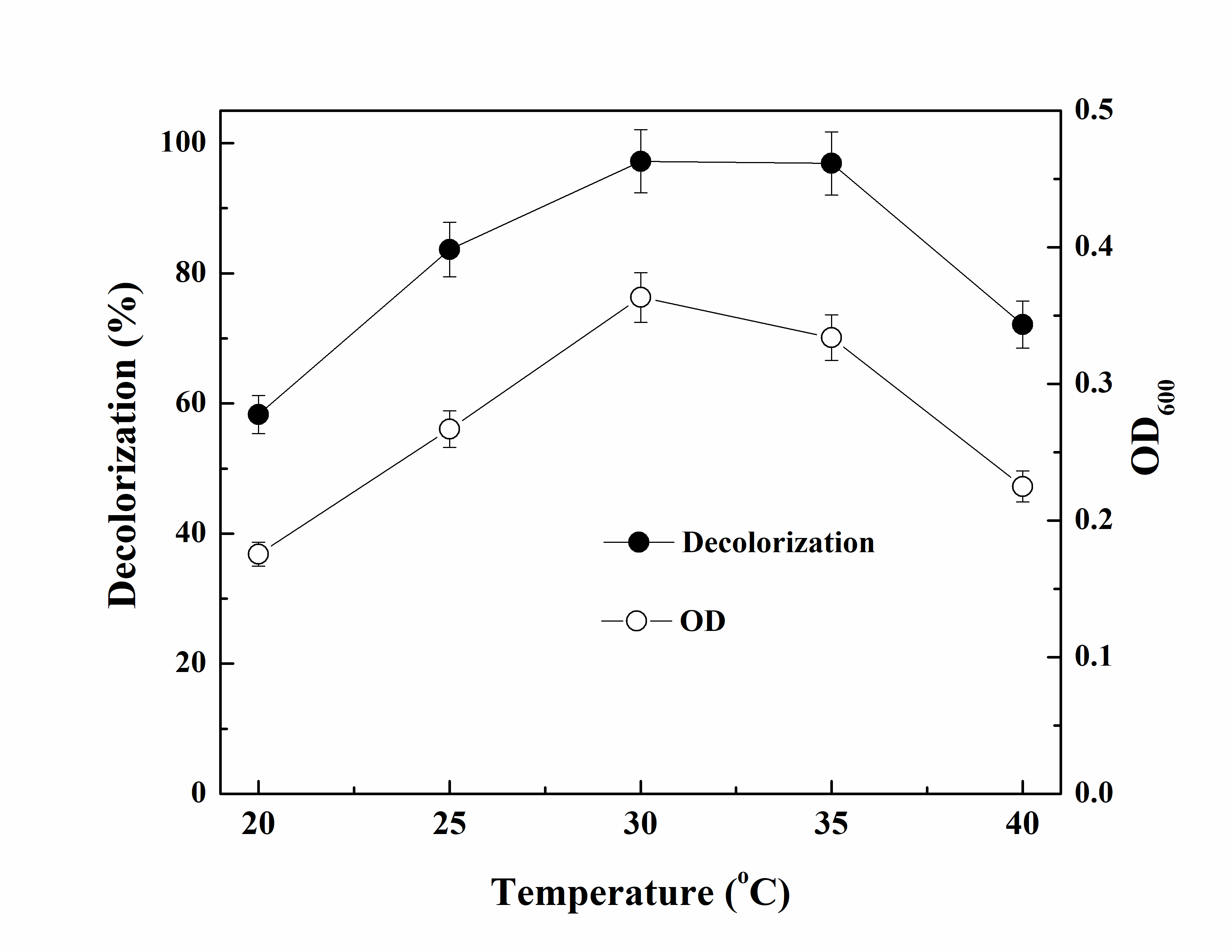
**

**(E) (F)**

**
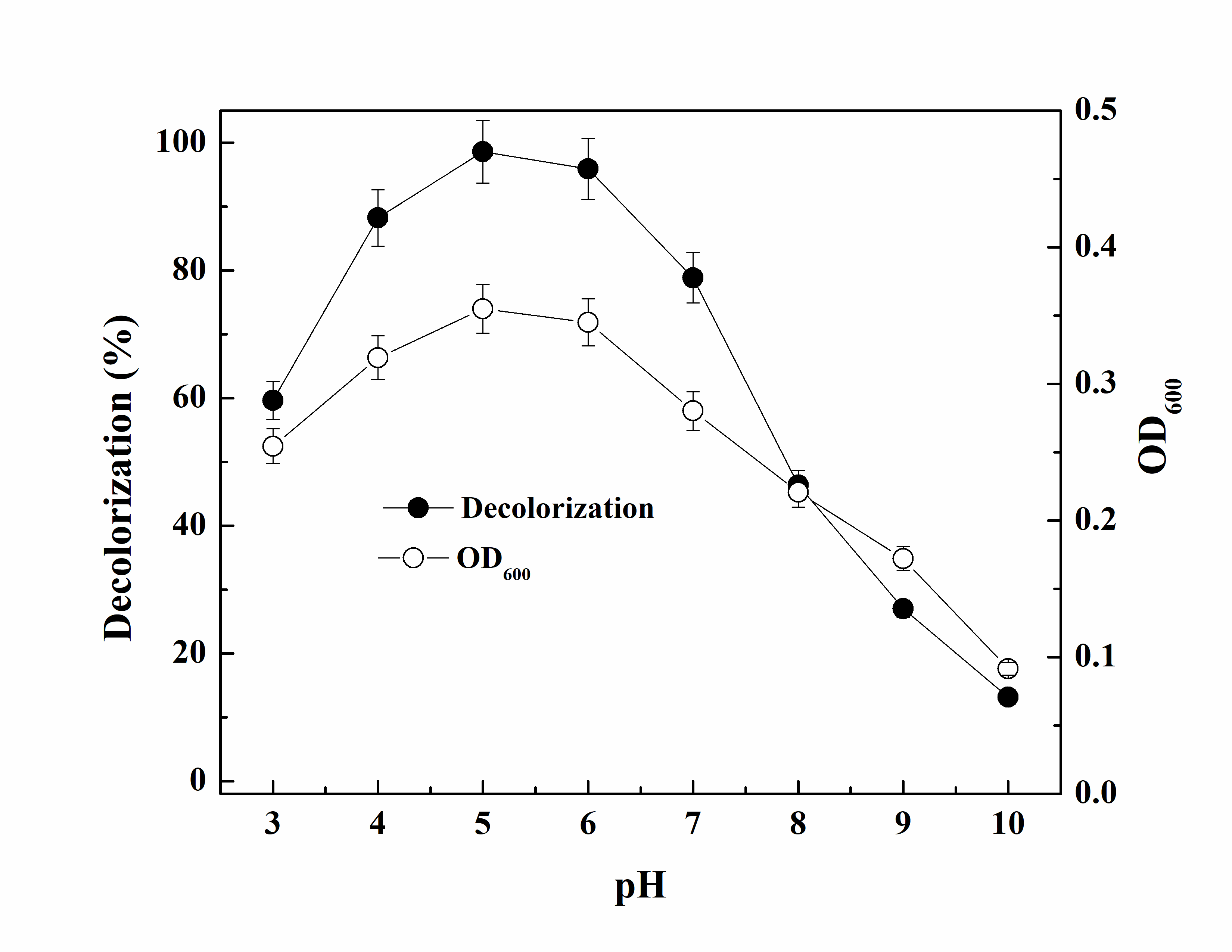
**

**(G)**

**Fig. S3** The effect of different parameters on decolorizing 80 mg/L ARB by growing cells of yeast A2 and cell growth (the OD_600_ values were analyzed after 10-fold dilution). A, glucose concentration; B, (NH_4_)_2_SO_4_ concentration; C, yeast extract concentration; D, NaCl concentration; E, rotation speed; F, temperature; G, pH.


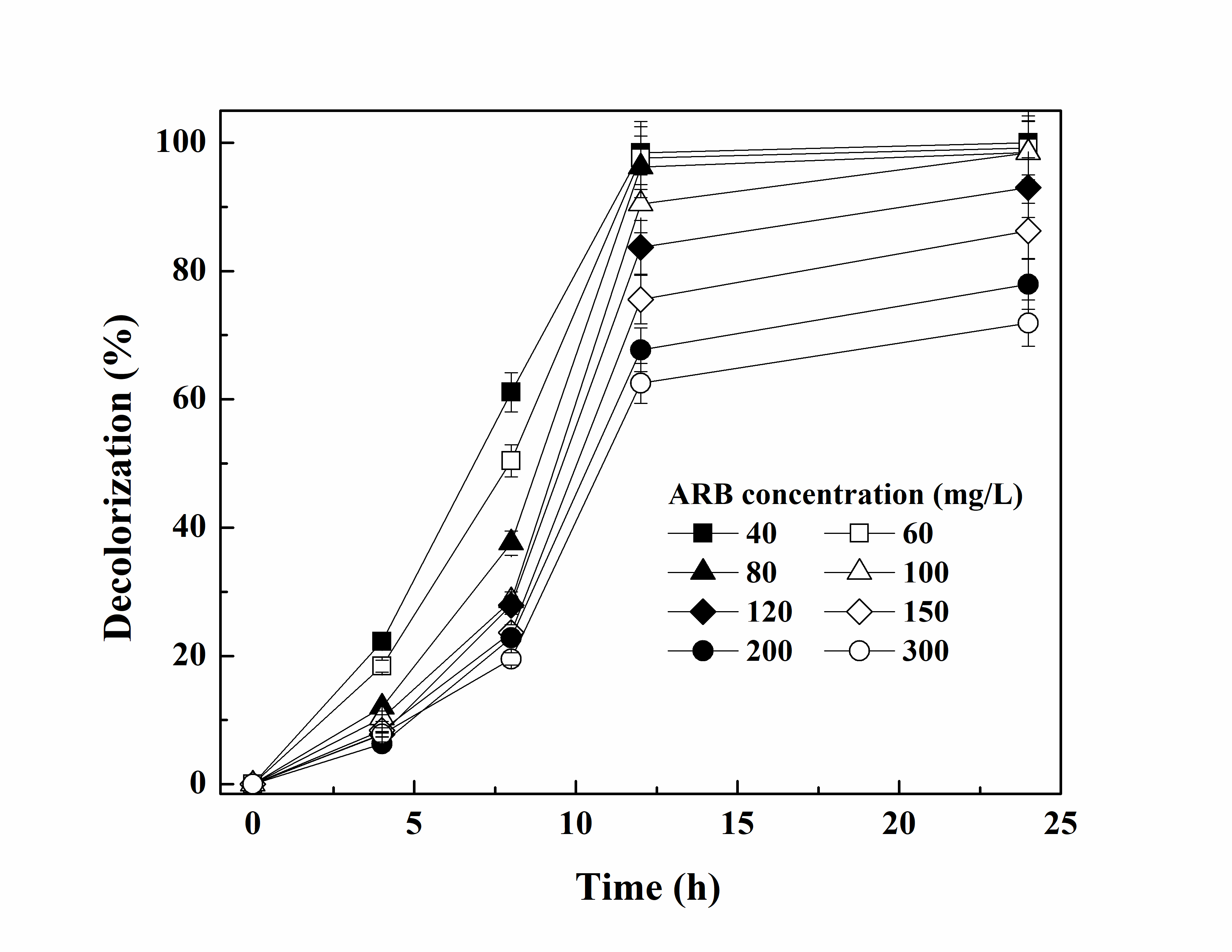

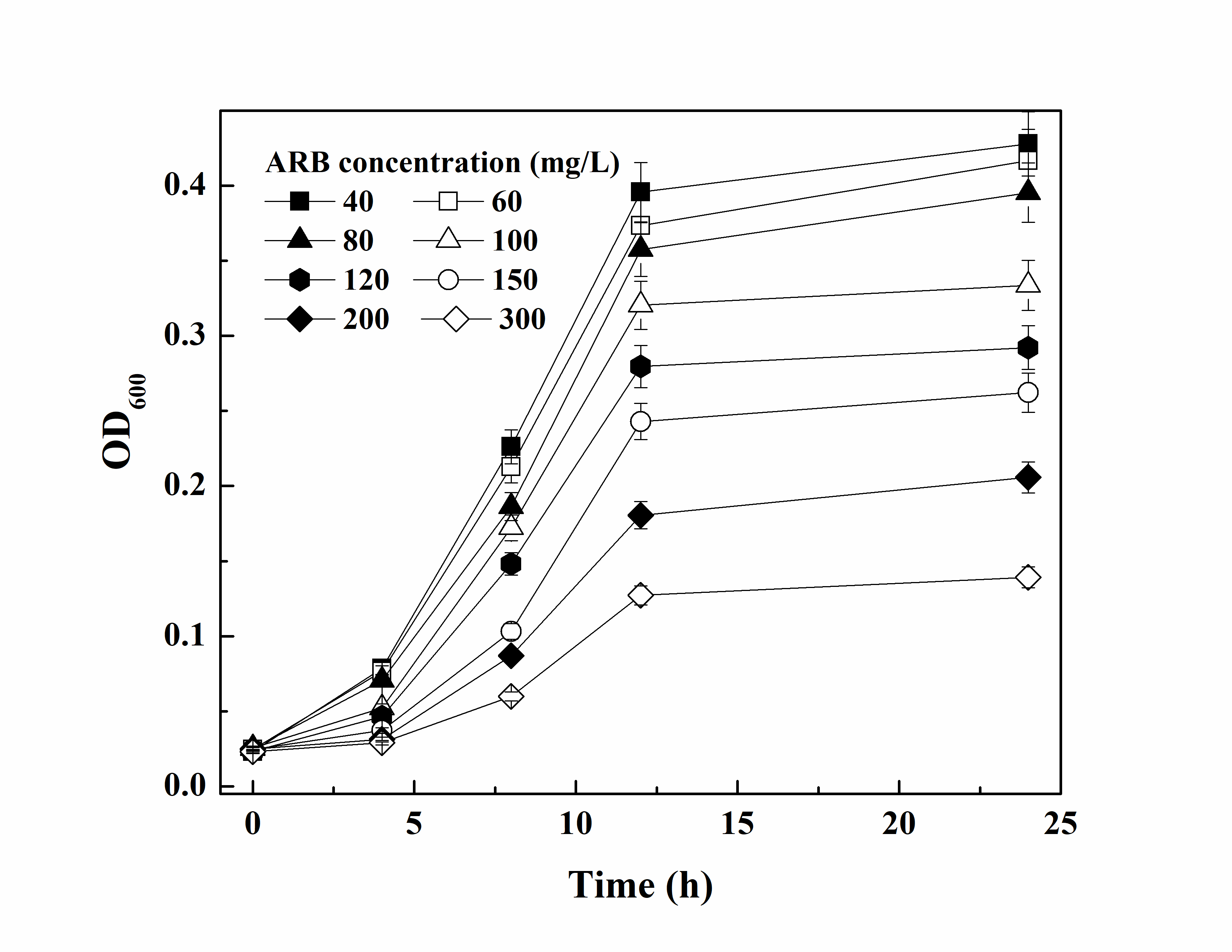


**(A) (B)**

**Fig. S4** The effect of initial ARB concentration on A, decolorization by growing cells of yeast A2 and B, its cell growth (the OD_600_ values were analyzed after 10-fold dilution).

**
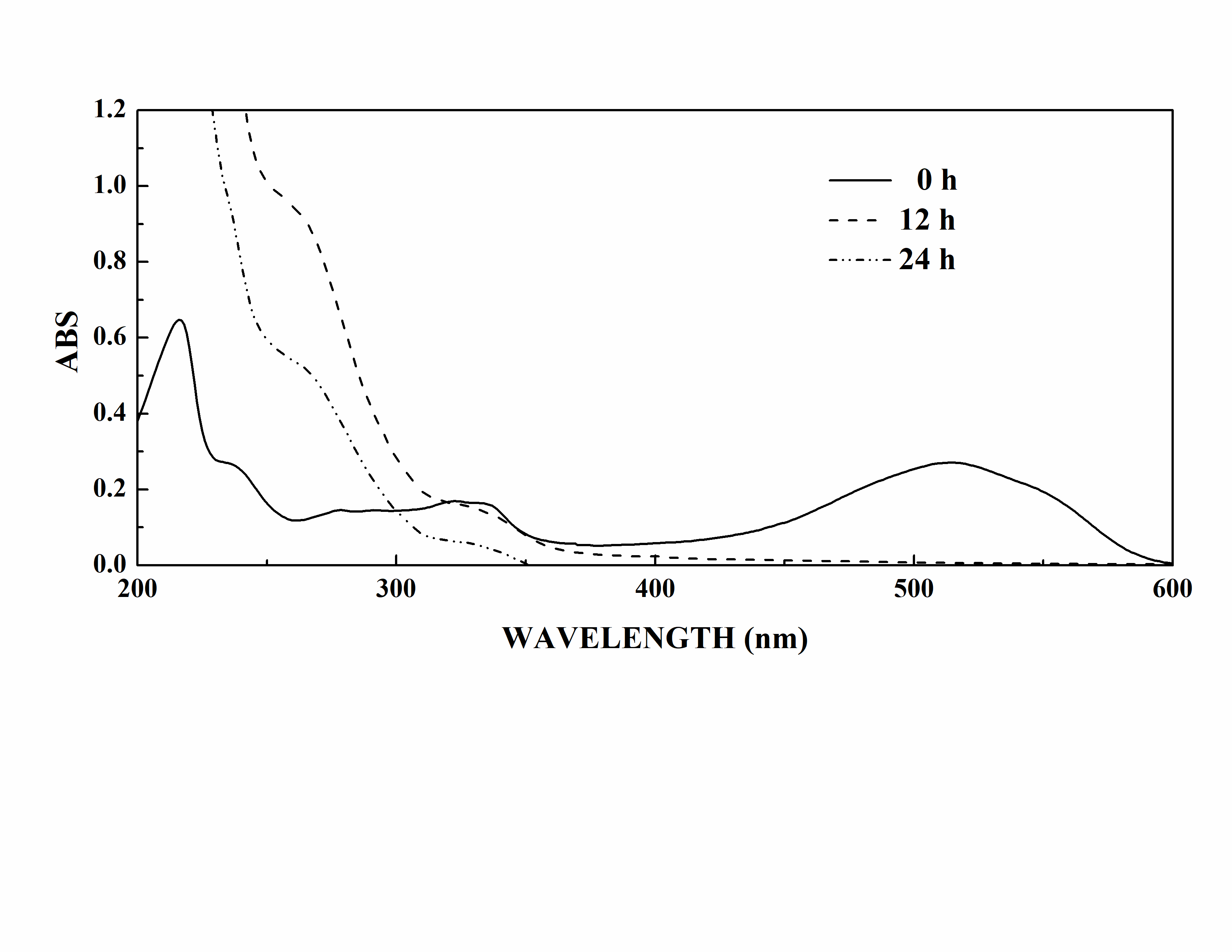
**

**(A)**

**I**

**II**

**III**

**VI**

**(B)**

**Fig. S5** A, UV-Vis spectra of 50 mg/L ARB before and after decolorization (for 12 h and 24 h) by growing cells of yeast A2. B, HPLC-MS spectra of intermediates formed from ARB decolorization. I, 4-hydrazinylnaphthalene-1-sulfonic acid; II, 4-hydroxynaphthalene-1-sulfonic acid; III, 4-aminonaphthalene-1-sulfonic acid; VI, naphthalene-1,2,4-triol.

**
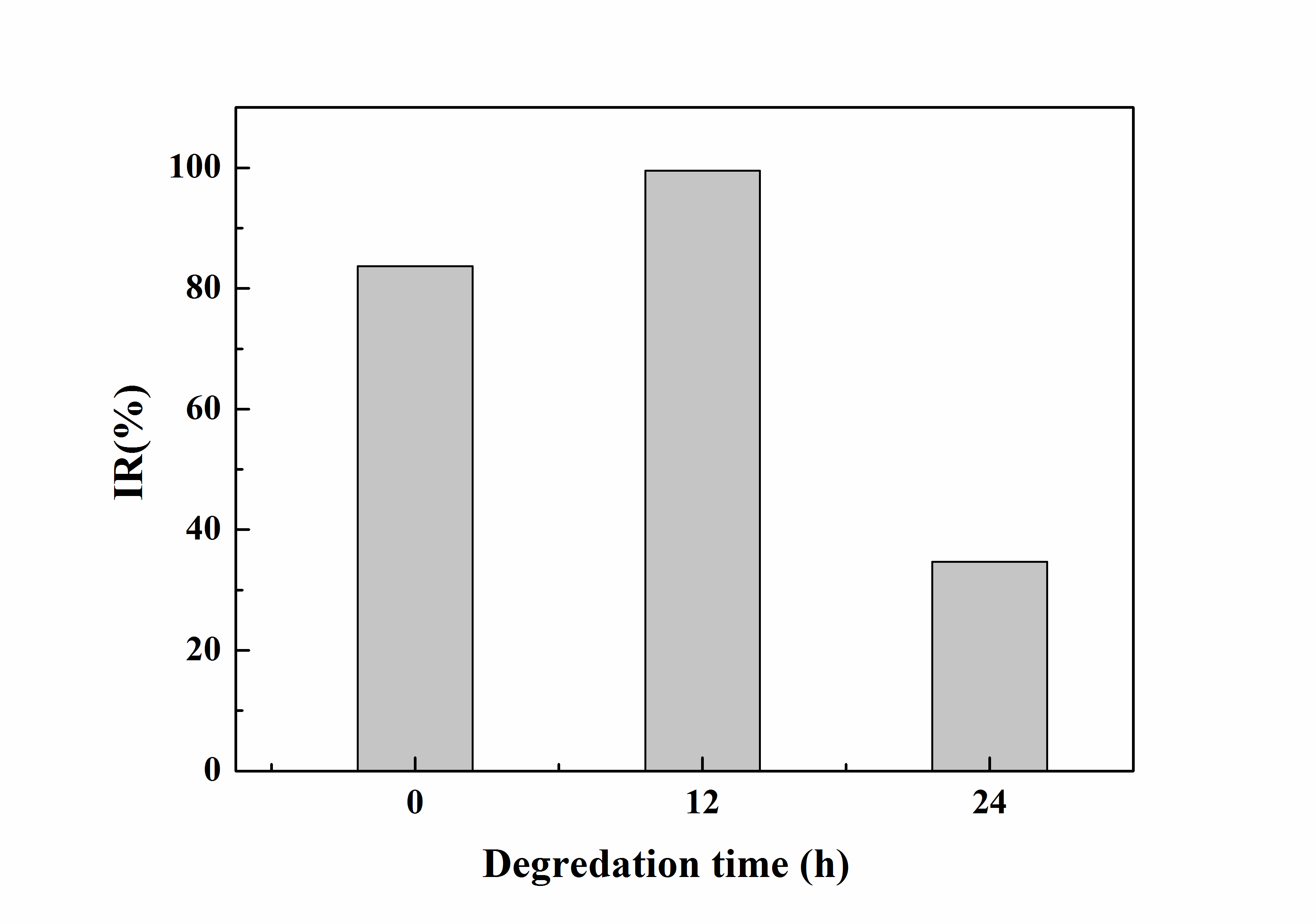
**

**Fig. S6** The acute toxicity of 80 mg/L ARB (with 30 g/L NaCl) before and after treatment by growing cells of yeast A2 for 12 h and 24 h, which were assessed by the Microtox test. 70 ≤ IR (%) < 100, high toxicity; 50 ≤ IR (%) < 70, moderate toxicity; 20 ≤ IR (%) < 50, low toxicity; 10 ≤ IR (%) < 20, micro toxicity; 0 ≤ IR (%) < 10, non-toxicity.

**
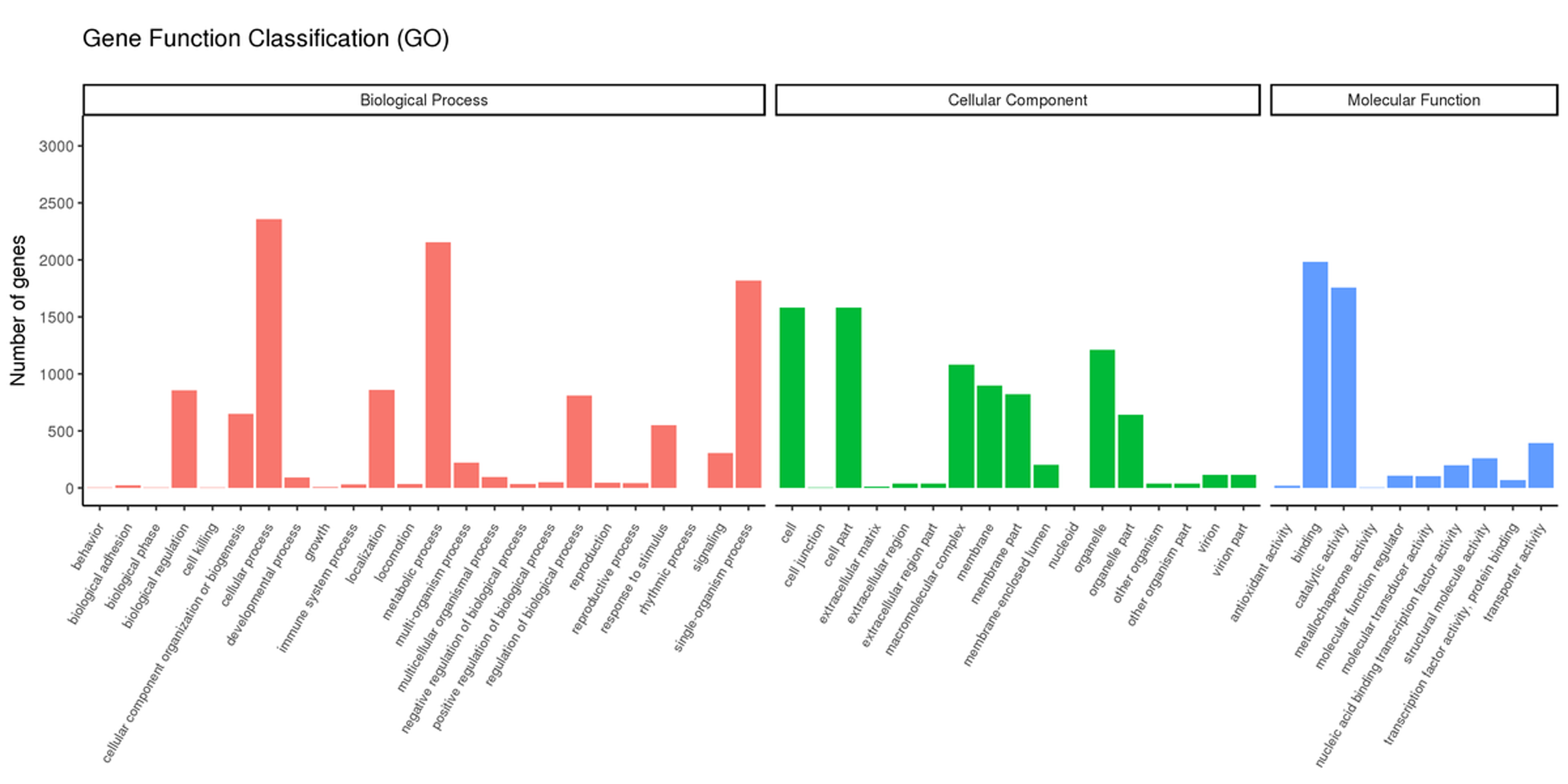
**

**(A)**

**
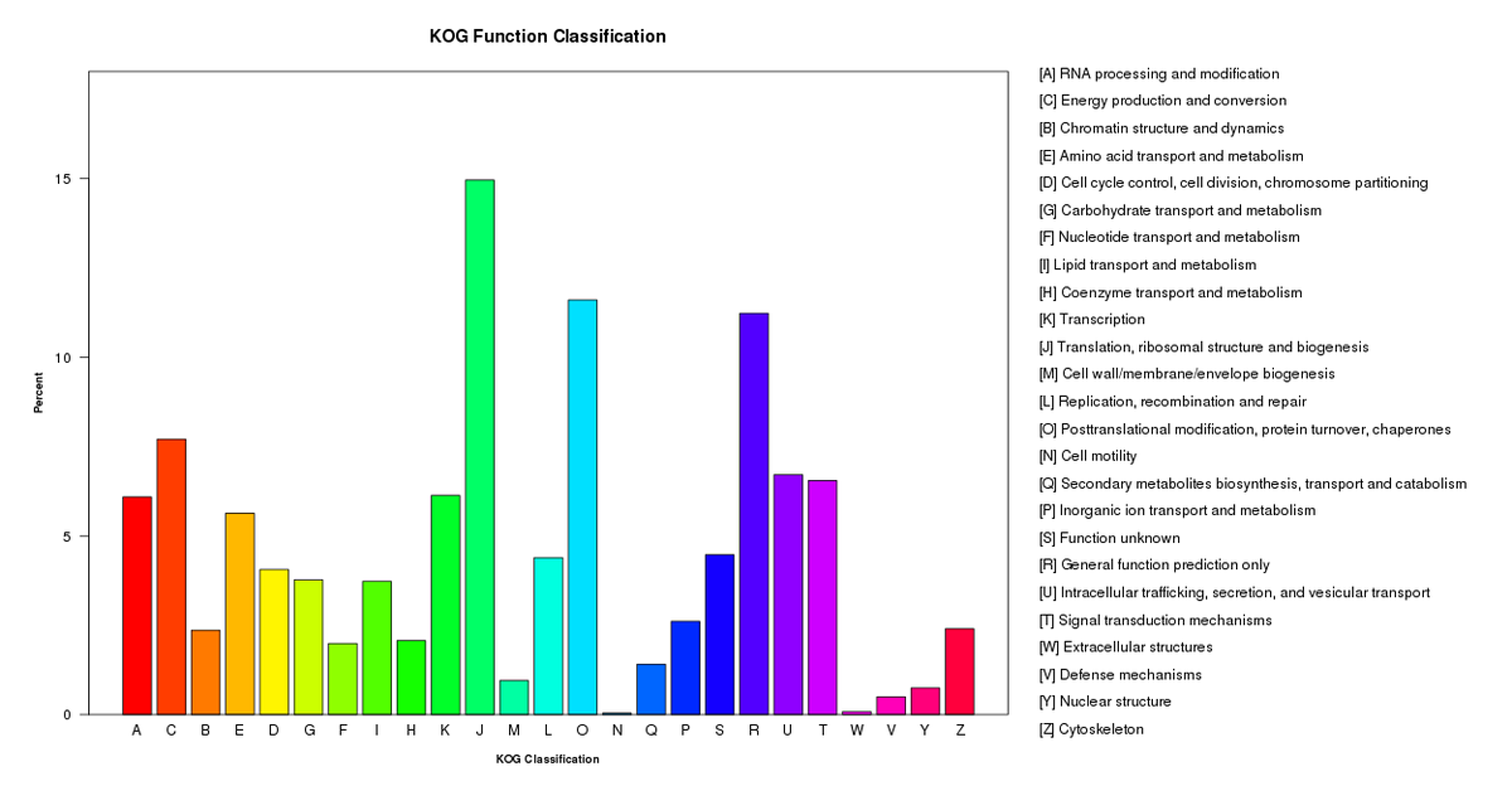
**

**(B)**

**
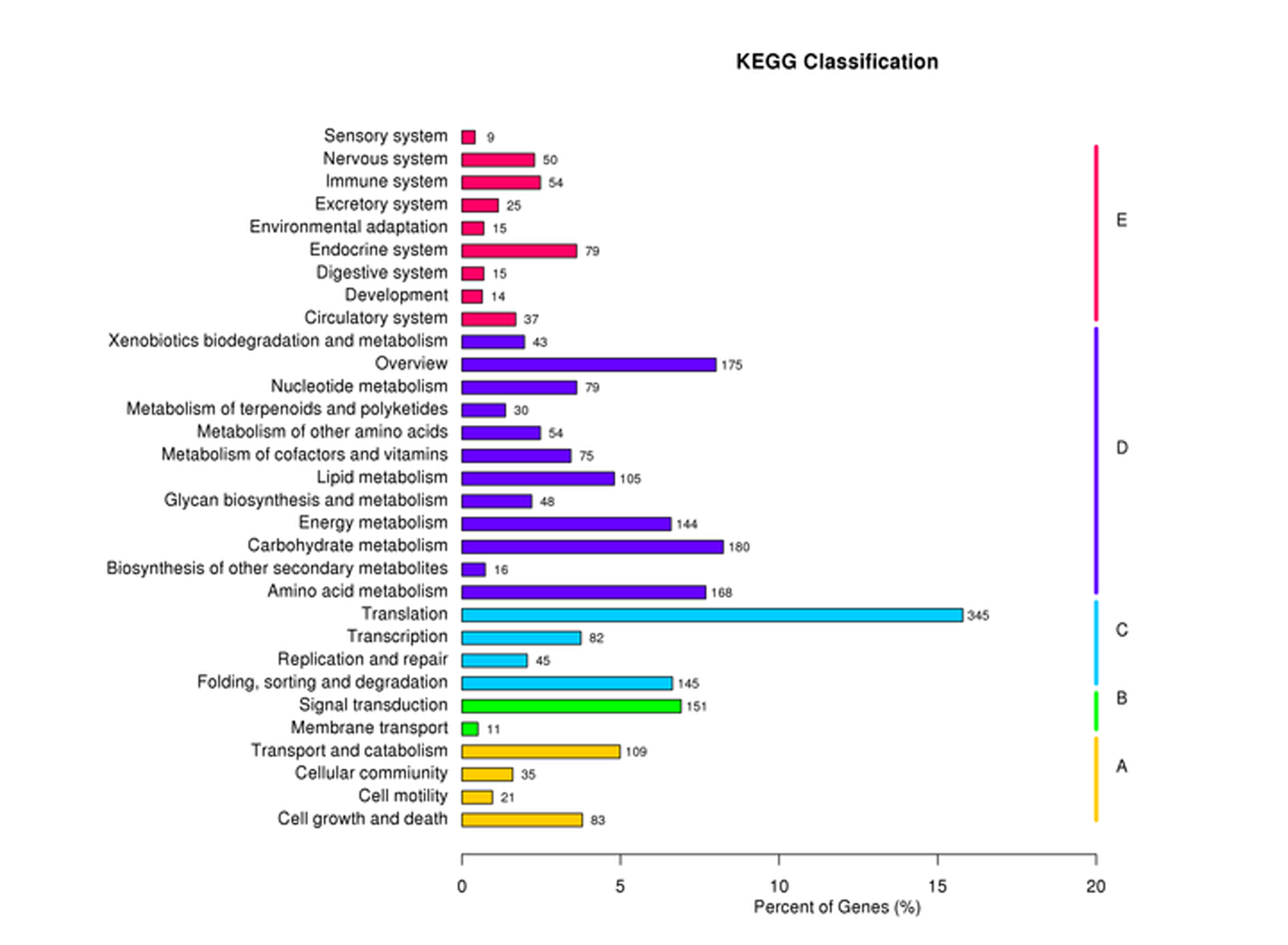
**

**(C)**

**Fig. S7** Gene classification diagram. A, GO classification diagram. B, KOG classification diagram. C, KEGG classification diagram. The ordinate is the name of the KEGG metabolic pathway, and the abscissa is the ratio of the number of genes annotated to the pathway and the total number of genes annotated. Genes are divided into five branches based on KEGG metabolic pathways (A, cellular processes; B, environmental information processing; C, genetic information processing; D, metabolism; E, organismal systems).
